# Reprogramming of epidermal keratinocytes by PITX1 transforms the cutaneous cellular landscape and promotes wound healing

**DOI:** 10.1172/jci.insight.182844

**Published:** 2024-12-20

**Authors:** Andrew M. Overmiller, Akihiko Uchiyama, Emma D Hope, Subhashree Nayak, Christopher G. O’Neill, Kowser Hasneen, Yi-Wen Chen, Faiza Naz, Stefania Dell’Orso, Stephen R. Brooks, Kan Jiang, Maria I. Morasso

**Affiliations:** 1 Laboratory of Skin Biology, National Institute of Arthritis and Musculoskeletal and Skin Diseases, NIH, Bethesda, Maryland, USA.; 2 Department of Genomics and Precision Medicine, George Washington University School of Medicine and Health Sciences, Washington, DC, USA.; 3 Center for Genetic Medicine Research, Children’s National Hospital, Washington, DC, USA.; 4 Genomic Technology Section and; 5 Biodata Mining and Discovery Section, National Institute of Arthritis and Musculoskeletal and Skin Diseases, NIH, Bethesda, Maryland, USA.

**Keywords:** Dermatology, Bioinformatics, Mouse models, Skin

## Abstract

Cutaneous wound healing is a slow process that often terminates with permanent scarring while oral wounds, in contrast, regenerate after damage faster. Unique molecular networks in epidermal and oral epithelial keratinocytes contribute to the tissue-specific response to wounding, but key factors that establish those networks and how the keratinocytes interact with their cellular environment remain to be elucidated. The transcription factor PITX1 is highly expressed in the oral epithelium but is undetectable in cutaneous keratinocytes. To delineate if PITX1 contributes to oral keratinocyte identity, cell-cell interactions, and the improved wound healing capabilities, we ectopically expressed PITX1 in the epidermis of murine skin. Using comparative analysis of murine skin and oral (buccal) mucosa with single-cell RNA-Seq and spatial transcriptomics, we found that PITX1 expression enhances epidermal keratinocyte migration and proliferation and alters differentiation to a quasi–oral keratinocyte state. PITX1^+^ keratinocytes reprogrammed intercellular communication between skin-resident cells to mirror buccal tissue while stimulating the influx of neutrophils that establish a pro-inflammatory environment. Furthermore, PITX1^+^ skin healed significantly faster than control skin via increased keratinocyte activation and migration and a tunable inflammatory environment. These results illustrate that PITX1 programs oral keratinocyte identity and cellular interactions while revealing critical downstream networks that promote wound closure.

## Abstract 2

Using scRNA-seq and Xenium spatial analysis, we illustrate that the oral epithelial transcription factor PITX1 transforms epidermal keratinocytes and the skin to promote wound healing.

## Introduction

Epithelial wound healing is a complex and tightly orchestrated biological process that restores tissue integrity and function after injury. Disparate body sites, such as the oral mucosa and skin, share a canonical cascade of healing stages after injury including (a) hemostasis, (b) inflammation, (c) reepithelialization/proliferation, and (d) tissue remodeling (1). Though similar in the overall progression of wound healing, the oral mucosa is known to heal faster than the skin and with higher fidelity (i.e., absence of or minimal scarring) and regeneration of adnexal structures, such as salivary glands (2). Multiple factors are known to influence the superior healing capabilities of the oral mucosa, including saliva, a measured inflammatory response to injury, and enhanced migratory and proliferative capabilities of the oral keratinocytes (3). Identifying the molecular factors that distinguish the 2 populations of keratinocytes has been a subject of ongoing investigation with the goal of applying insights to the design of novel therapeutic strategies for acute and chronic wounds (4).

Multiple comparative studies of oral and cutaneous wound healing have leveraged transcriptomic methodologies to identify the differences in healing progression (5). Earlier studies identified differences in the expression of immune-related genes during acute wound healing in oral and cutaneous tissues (3, 4, 6, 7). Notably, acute skin wounds express higher levels of collagens and enzymes related to tissue remodeling during the remodeling phase of healing, suggesting that mucosa rapidly resolves tissue remodeling while the skin is caught in a prolonged process of reorganization that leads to scarring. Follow-up studies demonstrated unique epithelial gene expression signatures in oral and skin acute wounds. They confirmed that oral keratinocytes migrate and proliferate much faster than skin-derived keratinocytes. Critically, oral keratinocytes release fewer inflammatory cytokines and chemokines both at baseline and when stimulated with inflammatory mediators, such as IL-1β or IL-6 (3, 6). Recently, a human clinical study allowed for longitudinal and paired wound healing sampling (4). RNA-Seq was used to compare the transcriptomes of human oral and skin acute wounds up to 6 days after injury. Confirming prior observations of enhanced healing and tightly regulated immune and epithelial responses, this study illustrated that the transcriptomic patterning of oral keratinocytes exists in a primed state that is more responsive to insult than the skin. Comparing the transcriptomes of the human acute wounds to published datasets of human and murine keratinocytes, a small subset of genes unique to oral keratinocytes and virtually absent in skin keratinocytes was identified and included the transcription factors SOX2 and PITX1. SOX2 is vital for the establishment and maintenance of stem cells in oral mucosa but is found only in the dermal papilla and Merkel cells in the adult skin (8, 9). Ectopic expression of SOX2 in murine epidermal keratinocytes led to enhanced wound healing via increased keratinocyte proliferation and secretion of EGFR ligands (10). The role of PITX1 in both oral keratinocyte identity and wound responsiveness has not been defined.

PITX1 is a homeodomain transcription factor that is critical for the embryonic development of hind limbs and the anterior pituitary gland and for establishing left-right asymmetry (11). In the role of regulating epithelial biology, PITX1, along with SOX2 and the epidermal differentiation regulator TP63, function in a ternary transcriptional complex to maintain tumor-propagating cells in cutaneous squamous cell carcinoma (SCC) (12). Including its identified role as an oral signature gene during wound healing, PITX1 also contributes to the differentiation of limbal stem/progenitor cells to corneal epithelial cells downstream of RAR-related orphan receptor A (RORA) activation and chromatin remodeling (13). We hypothesize that expression of PITX1 in adult oral and corneal epithelium and its absence in the epidermis suggests that it may play a key role in tissue-specific keratinocyte identity and the ability of the tissue to heal more rapidly following injury compared with the skin.

The present study generated an inducible transgenic mouse model ectopically expressing PITX1 in the epidermis to assess how the transcription factor affects keratinocyte identity and the process of wound healing. To compare tissue-specific cell-cell interactions and consequences of PITX1 expression, scRNA-Seq and Xenium in situ spatial transcriptomics were performed on whole adult skin from control and PITX1-expressing mice and buccal mucosa from control mice. Keratinocytes comprised most cells in all datasets and were more proliferative in both the PITX1 skin and buccal mucosa (PCNA^+^). Subpopulations of skin keratinocytes from PITX1 mice were shifted to a more oral-like transcriptomic state by expressing canonical activated keratinocyte and oral genes that were direct transcriptional targets of PITX1 as verified by CUT&Tag-Seq. Uniquely, PITX1 expression stimulated pro-inflammatory gene expression networks, recruiting neutrophils to the skin of these mice. These neutrophils were highly concentrated around hair follicles and had distinct cell-cell communication with various subpopulations in the PITX1 skin. Finally, PITX1 enhanced full-thickness cutaneous wound healing by promoting keratinocyte migration and stimulating a neutrophil-spearheaded immune response into the wound bed. This study demonstrates that PITX1 is a key transcriptional determinant of oral keratinocyte identity and can independently drive a pro-healing wound response. The combination of single-cell and spatial transcriptomic analyses identified key factors driving the skin-to-oral keratinocyte lineage shift and neutrophil recruitment that may serve as targets for therapeutic modalities to treat cutaneous wounds.

## Results

### Generation and histologic analysis of PITX1^+^ mice.

**Figure 1:**
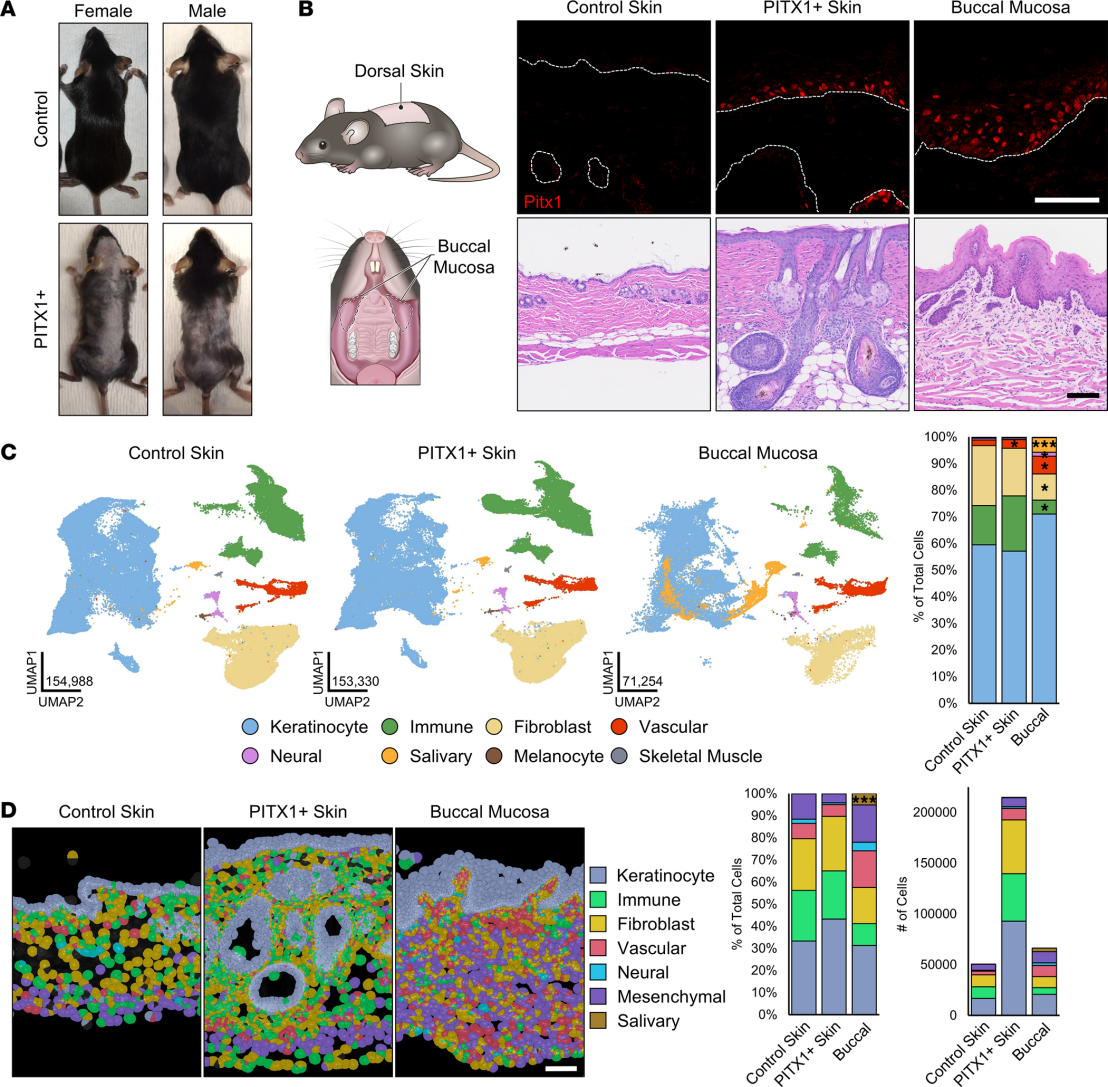
scRNA-Seq and Xenium in situ analysis of control skin, PITX1^+^ skin, and buccal mucosa. (**A**) Representative dorsal images of male control and PITX1^+^ male and female mice. (**B**) Schematic of tissues collected (left). Representative immunofluorescent (IF) stainings for PITX1 (shown in red) and DAPI (shown in blue) in control epidermis, PITX1^+^ epidermis, and buccal epithelium. Dotted lines denote dermal-epidermal junction (DEJ) (top, middle). Representative H&E of tissues (bottom). Scale bars = 100 μm. (**C**) Uniform manifold approximation and projections (UMAPs) and cell type annotations of whole skin scRNA-Seq (left) and proportion plot of cell types in each condition (right). *n* = 8 control mice, 8 PITX1^+^ mice, 7 buccal mucosae pools. (**D**) Xenium in situ representative images of male FFPE skin sections with Xenium-derived cell types highlighted. *n* = 3 control skin, 4 PITX1^+^ mice, 7 buccal mucosae; scale bar = 100 μm (left). Proportion plot and total number of each cell type in each condition (right). Significance for proportion plots was assessed by proportionality testing followed by ad hoc comparisons against the corresponding cell type in control skin to derive log_2_ fold-change (log_2_FC) (*P < 0.01 & log_2_FC > |1.5|, ***P < 0.01 & log_2_FC > |4|).

To investigate a possible role of the transcription factor PITX1 in programming an oral epithelial lineage, we generated Tet-On, TRE-*Pitx1*/*Krt5*-rtTA transgenic mice (14). Littermates were fed either normal chow (control) or doxycycline-containing chow (PITX1^+^) starting in telogen hair phase for 6 weeks to induce PITX1 expression in the epidermis (Supplemental Figure 1A; supplemental material available online with this article; https://doi.org/10.1172/jci.insight.182844DS1) (15). PITX1 expression in the skin caused extensive, irreversible alopecia that primarily affected the dorsal and ventral skin but occasionally spread to occipital and peripheral regions as well (Figure 1A and Supplemental Figure 1A). We verified ectopic PITX1 expression in the skin via immunofluorescence (IF) staining and quantitative PCR (qPCR) and showed endogenous PITX1 expression in control mouse buccal mucosa (Figure 1B and Supplemental Figure 1B). Histologically, PITX1^+^ skin had extensive epidermal and follicular hyperplasia and parakeratosis, sebaceous gland hyperplasia, and substantial immune cell infiltration into both the dermis and surrounding the hair follicles, suggesting a breakdown of the immunoprivilege of the follicles (Figure 1B) (16). The hypertrophy of the hair follicles in the PITX1^+^ mice corresponds with follicles arrested at the peak of the anagen. Comparatively, the hyperplastic interfollicular epidermis mirrored the much thicker epithelium of the buccal mucosa (Figure 1B). Oral keratinocytes in vivo proliferate much faster than their cutaneous counterparts (3). Both PITX1^+^ skin and buccal mucosa had significantly increased proliferation in vivo as measured by PCNA IF (Supplemental Figure 1C). Another feature of oral keratinocytes is their enhanced migratory potential compared with cutaneous keratinocytes (4). Similarly, PITX1^–^ expression significantly drove skin keratinocyte migration in vitro (Supplemental Figure 1D). Together, PITX1^+^ murine skin has structural and functional changes in the keratinocyte populations, such as increased proliferative and migratory capabilities.

### Comparative single-cell RNA-Seq and Xenium in situ analysis of control skin, PITX1^+^ skin, and buccal mucosa.

While PITX1^+^ expression is limited to keratinocytes, extensive alterations in other skin-resident cells were readily apparent upon transgene induction (Figure 1, A and B). To evaluate the effect of PITX1 expression in all compartments of the skin and compare those changes with healthy buccal mucosa, we performed single-cell RNA-Seq (scRNA-Seq) and Xenium in situ spatial analysis (Figure 1, C and D). The cells obtained from control skin (*n* = 8 mice), PITX1^+^ skin (*n* = 8), and buccal mucosa (*n* = 7 pools) for sequencing and analysis were pre-processed and screened for quality control features prior to bioinformatic integration using the Seurat platform and manual annotation of resultant clusters to generate generic cell type categories (Supplemental Figure 2, A–E, and Supplemental Methods) (17, 18). Across the conditions, most cells were keratinocytes (57%–71%) while immune cells (5%–21%) and fibroblasts (10%–22%) comprised most of the remaining cells in the skin sets, and vascular (2%–7%) and salivary gland cells (0%–6%) comprised high proportions of the buccal mucosa set (Figure 1C). Minor cell types, such as neural cells (0.6%–1.4%), melanocytes (0%–0.21%), and skeletal muscle cells (0.12–0.23%), were also captured in this analysis. The buccal mucosa had fewer immune cells and fibroblasts and more vascular and salivary cells compared with control skin. The increased proportion of salivary cells in the buccal mucosa was likely an artifact of a single buccal mucosa sample having a high number of these cells (Supplemental Figure 2B). Intriguingly, PITX1^+^ skin has a slightly, but significantly, increased proportion of vascular cells while having an increased, but nonsignificant, proportion of immune cells (Figure 1C). These variabilities are a result of the slightly different proportions of cells captured between sexes in the 3 tissue types — PITX1^+^ females had significantly more immune cells than control females while PITX1^+^ males had significantly more vascular cells (Supplemental Figure 2C). Additionally, a few (470 control, 775 PITX1^+^) skin cells clustered with salivary cells. Analyzing where these “salivary” cells clustered in a skin-only dataset, the majority were keratinocytes, though some also clustered with immune cells, fibroblasts, and melanocytes (Supplemental Figure 2D). These results illustrate the complexity and challenge of integrating single cells isolated from different tissues that other studies have also confronted (19).

Though scRNA-Seq allows powerful insight into the transcriptomics of individual cells constituting a tissue, it inherently lacks the capability to discern organization and precise cell-cell interactions. To address this, we performed Xenium in situ spatial analysis on skin and buccal samples. Xenium allows the capture of precise subcellular localization of selected transcripts and algorithmically partitions cell borders, allowing the transcripts to be assigned to unique cells (20). Xenium was able to capture and distinguish the various unique cell types comprising both the skin and buccal mucosa with a custom panel of 328 transcript-targeting probes (Figure 1D; Supplemental Figure 3, A–C; and Supplemental Table 1). In total 331,572 cells were analyzed, and stark differences in the proportionality of the constituent cell types were found via Xenium: keratinocytes (31%–43%), immune cells (10%–23%), fibroblasts (16%–25%), and vascular (5%–17%), neural (0.8%–4%), mesenchymal (4%–17%), and salivary gland cells (0%–5%) (Figure 1D). These results likely reflect the real distribution of the various cell types as some cell types have been difficult to capture and sequence via scRNA-Seq (17, 21, 22). Insufficient samples were present to statistically evaluate any sex-based differences, though increases in keratinocytes, immune cells, and fibroblasts were observed upon PITX1 expression in both sexes (Supporting Data Values). Even so, cellular density of the PITX1^+^ skin was significantly higher than control skin, but not buccal mucosa, corroborating histologic findings of cellular influx to that tissue (Supplemental Figure 3D). Less common cell types of the skin (melanocytes, adipocytes, interstitial fibroblasts, Merkel cells, etc.) were not detected in the Xenium data because of the limited number of marker genes employed. Nevertheless, our scRNA-Seq and Xenium data sets allow for more refined interrogation of the cellular interactions underpinning tissue specificity.

### PITX1 reshapes the identity of epidermal keratinocytes.

**Figure 2:**
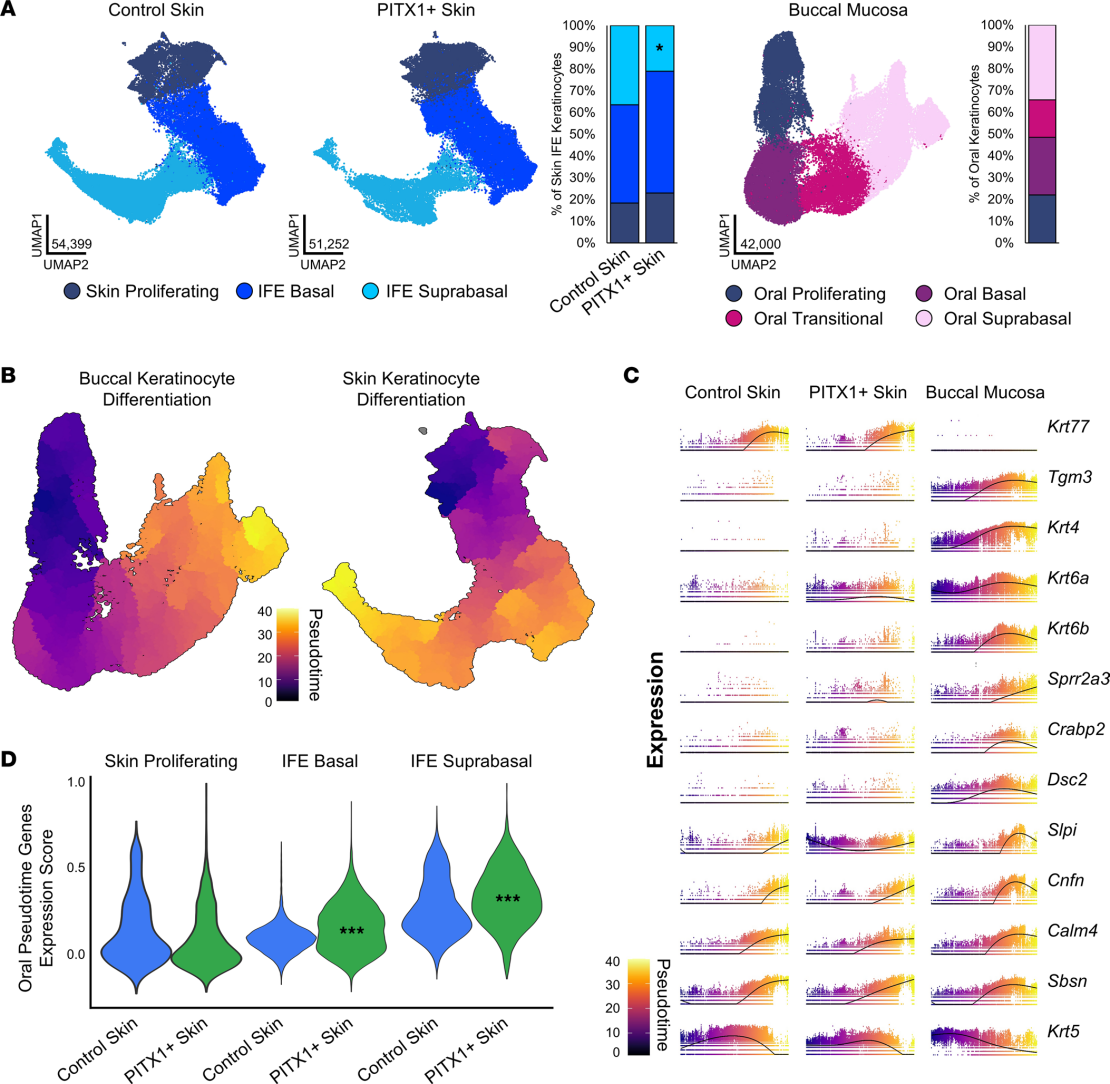
PITX1 reprograms epidermal interfollicular keratinocyte subtypes. (**A**) UMAPs of control skin and (*n* = 8) PITX1^+^ (*n* = 8) interfollicular epidermal (IFE) keratinocyte subpopulations and proportion plot (left). UMAP of buccal keratinocyte (*n* = 7) subpopulations and proportion plot (right). Significance for skin proportion plot was assessed by proportionality testing followed by ad hoc comparisons against the corresponding cell type in control skin to derive log_2_ fold-change (log_2_FC) (*P < 0.01 & log_2_FC > |1.5|). (**B**) Pseudotime trajectory plots of buccal (left) and integrated IFE keratinocyte data sets (right). (**C**) Plots illustrating gene expression as a function of pseudotime of individual cells (dots) in each condition. Line indicates average gene expression over pseudotime. (**D**) Violin plots of the relative expression of the top oral differentiation-associated genes in skin IFE subpopulations. Data are shown as the distribution of relative expression scores for individual cells for each condition. Significance in each skin subpopulation was determined by pairwise Wilcoxon’s rank sum tests with Benjamini-Hochberg multiple comparisons correction (***P < 0.001).

To assess if cutaneous PITX1 expression affects keratinocyte homeostasis and differentiation, skin and oral keratinocytes were subsetted separately and annotated based upon gene expression (Figure 2A and Supplemental Figure 4, A and B). Whereas the skin sample keratinocyte populations were roughly divided between IFE and hair follicle (HF) populations (control: 48% IFE, 37% HF; PITX1^+^: 45% IFE, 39% HF), oral keratinocytes were only divided on stratified epithelial subtypes, reflecting the lack of hair follicles. In the skin, PITX1 expression drove the expansion of Anagen HF and Sebaceous keratinocytes while reducing the proportions of HF Stem Cell, Upper HF Basal, and IFE Suprabasal populations (Supplemental Figure 4B). Morphologic observation of the hypertrophic PITX1^+^ hair follicles and sebaceous glands corroborated the scRNA-Seq findings (Figure 1B). The HF keratinocytes from the skin-only keratinocyte dataset were further subsetted (Supplemental Figure 4C). Given the apparent diminution in the proportion of HF-renewing stem cells and concomitant increase in terminally differentiated anagen HF keratinocytes with PITX1 expression, Ingenuity Pathway Analysis (IPA) was performed on the set of differentially expressed genes between control and PITX1^+^ populations (Supplemental Figure 4D). PITX1^+^ Anagen HF cells showed activation of proliferation, growth, and hyperplasia ontologies while the HF Stem Cells had immune response, tumor-related, and fibrosis/death-related ontologies activated. This analysis suggests that one potential mechanism underpinning the alopecia observed in these mice might be an immunologic assault on the HF Stem Cell population, leading to their decline and allowing Anagen HF cells to expand and ultimately terminally differentiate into a noncycling, nonfunctional HF. Indeed, Xenium analysis of hair follicles in PITX1^+^ skin corroborated this loss of HF Stem Cells while the proportion of terminally differentiating Anagen HF keratinocytes (evidenced by the expression of markers of terminal differentiation such as *Dlx3* and *Dsg4*) massively increased, leading to HF hypertrophy (Supplemental Figure 4, E and F).

### Cutaneous keratinocyte differentiation acquires oral characteristics following PITX1 expression.

Pseudotime analysis of scRNA-Seq data allows for the capture of gene regulation along continuous biological processes and cell types, including epithelial differentiation (23–25). Oral and skin IFE keratinocytes undergo similar processes of terminal differentiation as evidenced by pseudotime analysis with the Monocle3 package (Figure 2B). Critically, pseudotime analysis allows for assessing the continuum of gene expression along pseudotime and agnostic of precise cell subtype. First, the top 100 genes whose expression most strongly correlates with pseudotime in the oral keratinocytes were identified. Next, the expression of these genes during pseudotime in the skin IFE keratinocytes was assessed. Certain pseudotime-expressed genes, such as *Krt77* or *Tgm3*, were specific to skin or buccal mucosa, respectively, irrespective of PITX1 expression (Figure 2C). Crucially, oral differentiation-specific genes, such as *Krt4*, *Crabp2*, *Krt6a/b*, *Sprr2a3*, and *Dsc2*, were induced following PITX1 expression. *Krt6a/b* defined a subset of “activated” keratinocytes that are more migratory, proliferative, and pro-inflammatory, often in response to wounding, and are expressed at baseline in oral mucosa (26). Our previous study identified the human orthologs *SPRR2A* and *DSC2* as highly upregulated genes in human buccal mucosa compared with skin at baseline and during wound healing (4). *Krt4*, a differentiating oral keratinocyte-specific keratin, and *Crabp2*, responsible for shuttling retinoic acid (RA) to cell nuclei and highly expressed in oral keratinocytes, were both induced in PITX1^+^ skin during keratinocyte differentiation (27). The transcription factors comprising the oral signature set identified from this study were found to be expressed in oral keratinocytes during pseudotime but were absent from either control or PITX1^+^ skin, demonstrating PITX1 expression alone does not influence the expression of these other transcription factors in healthy skin (Supplemental Figure 5A). Expression of additional transcription factor genes previously identified to be regulated by PITX1, *Trp63* and *Klf4*, were unaffected in the PITX1^+^ condition as well (12). Other pan-epithelial genes, such as *Cnfn*, *Sbsn*, and *Krt5*, all had relatively equivalent expression over pseudotime across conditions. Next, every skin IFE keratinocyte was scored on the expression of the top 100 oral pseudotime-dependent genes, with a higher oral pseudotime gene expression score demonstrating higher expression of the set of oral genes (Figure 2D). Overall, PITX1^+^ keratinocytes had higher oral pseudotime gene expression scores, further increasing as they differentiated, supporting the key role in driving oral epithelial identity and oral keratinocyte differentiation by PITX1.

### CUT&Tag-Seq reveals genomic binding of PITX1.

**Figure 3:**
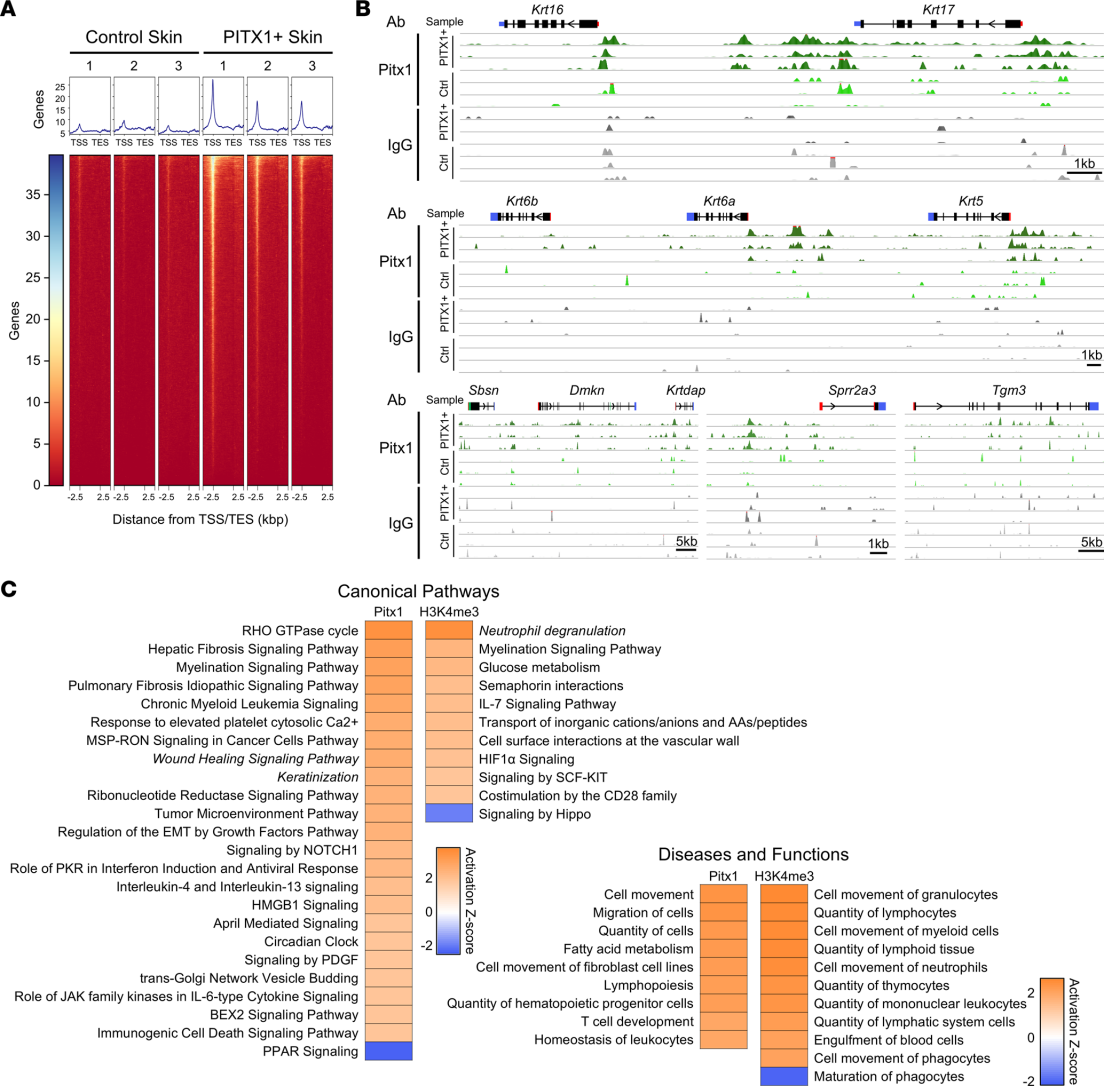
CUT&Tag-Seq of PITX1 illustrates binding to activated and oral keratinocyte genes. (**A**) Control (*n* = 3) and PITX1^+^ (*n* = 3) epidermal cell CUT&Tag-Seq heatmaps of average genomic occupancy of PITX1 over transcriptional units, starting 2.5 kb upstream of TSS and ending 2.5 kb downstream of transcription end sites. (**B**) Representative genomic tracks of control and PITX1^+^ epidermal CUT&Tag-Seq for PITX1 and IgG antibodies. Gene 5′-UTR (red bar), exons (black bars), alternatively spliced exons (green bars), 3′-UTR (blue bars), and direction of transcription (arrows) represented above tracks. (**C**) IPA ontologies of genes differentially occupied by PITX1 or histone H3K4me3 in PITX1^+^ mouse epidermis. *Z* score > |2| was considered significantly activated or repressed.

To determine which genomic loci are specifically bound by PITX1 in our mouse model, we employed CUT&Tag-Seq — a highly efficient and selective technique to assess genomic occupancy of histones or transcription factors like ChIP-Seq (28). CUT&Tag-Seq has superior specificity (i.e., lower background signal) and uses orders of magnitudes fewer cells as an input (roughly millions of cells for ChIP-Seq compared with 100,000 cells for CUT&Tag-Seq). The same workflow that produces single-cell suspensions for scRNA-Seq was adapted here to produce single cells for CUT&Tag-Seq. PITX1^+^ binding was greatest in the region of transcription start sites (TSS) (Figure 3A). Additional binding sites were found in regions proximal to the TSS — 5′ untranslated regions (5′-UTRs), first introns, and first exons. In parallel, H3K4 trimethylation (H3K4me3) CUT&Tag-Seq, which marks open chromatin at the promoter region, illustrated characteristic peak binding immediately upstream of and at the TSS (Supplemental Figure 6A). The activated keratinocyte genes *Krt6a*, *Krt16*, and *Krt17* all had significant enrichment of both PITX1 and H3K4me3 at the TSS, correlating with the increased transcription of these genes (Figure 3B and Supplemental Figure 6B). *Krt6b* itself had no proximal PITX1 peaks but was mildly enriched for H3K4me3, suggesting that PITX1 may function distally. Additional genes, such as *Krt5*, *Sbsn*, *Dmkn*, *Krtdap*, *Sprr2a3*, and *Tgm3*, had specific, proximal binding of PITX1 but no concomitant change in H3K4me3 occupancy, demonstrating that PITX1 may not directly regulate the transcription of these genes. HOMER motif analysis of PITX1 peaks found the canonical TAATCC binding motif in both intergenic and promoter-proximal region peaks (Supplemental Figure 6C) (29). Furthermore, intergenic PITX1 peaks were enriched for binding to a variety of KLF isoform motifs while promoter region PITX1 peaks bound many transcription factors with analogs of the TAATCC binding motif. No PITX1 binding motifs were found in IgG samples, demonstrating the specificity of PITX1 CUT&Tag-Seq (Supporting Data Values). To determine transcriptional networks directly modified by PITX1, we employed DESeq2 to find genes differentially bound by PITX1 in PITX1^+^ versus control epidermal cells (30). Using IPA, we found that PITX1 promoted the expression of genes that activate various injury-related and cellular signaling pathways (Figure 3C). Importantly, both wound healing signaling and keratinization pathways were enriched canonical pathway ontologies, and most of the activated diseases and functions ontologies were related to cellular movement. Performing the same analysis with genes that were enriched with H3K4me3 peaks after PITX1 expression, most ontologies in both categories were related to immune cell signaling or migration. The influx of immune cells into PITX1^+^ skin suggests intercellular signals arising from the keratinocytes that promote immune cell homing. Both *Tnf* and *Csf2* genes, potent immunostimulatory cytokines, had H3K4me3 enrichment in PITX1^+^ skin (Supplemental Figure 6B). The majority of PITX1 peaks overlapped with the active enhancer histone markers acetylated H3K27 (42.3%), the active promoter marker H3K4me3 (9.0%), or heterochromatin (H3K27me3; 4.6%), suggesting PITX1 is specifically binding genomic regions actively being transcribed or regulating genes that influence the noted ontologies (Supplemental Figure 6D). These data suggest PITX1 profoundly alters keratinocyte biology but may also have paracrine effects on tissue homeostasis.

### Fibroblast subtypes are altered by PITX1 expression.

**Figure 4:**
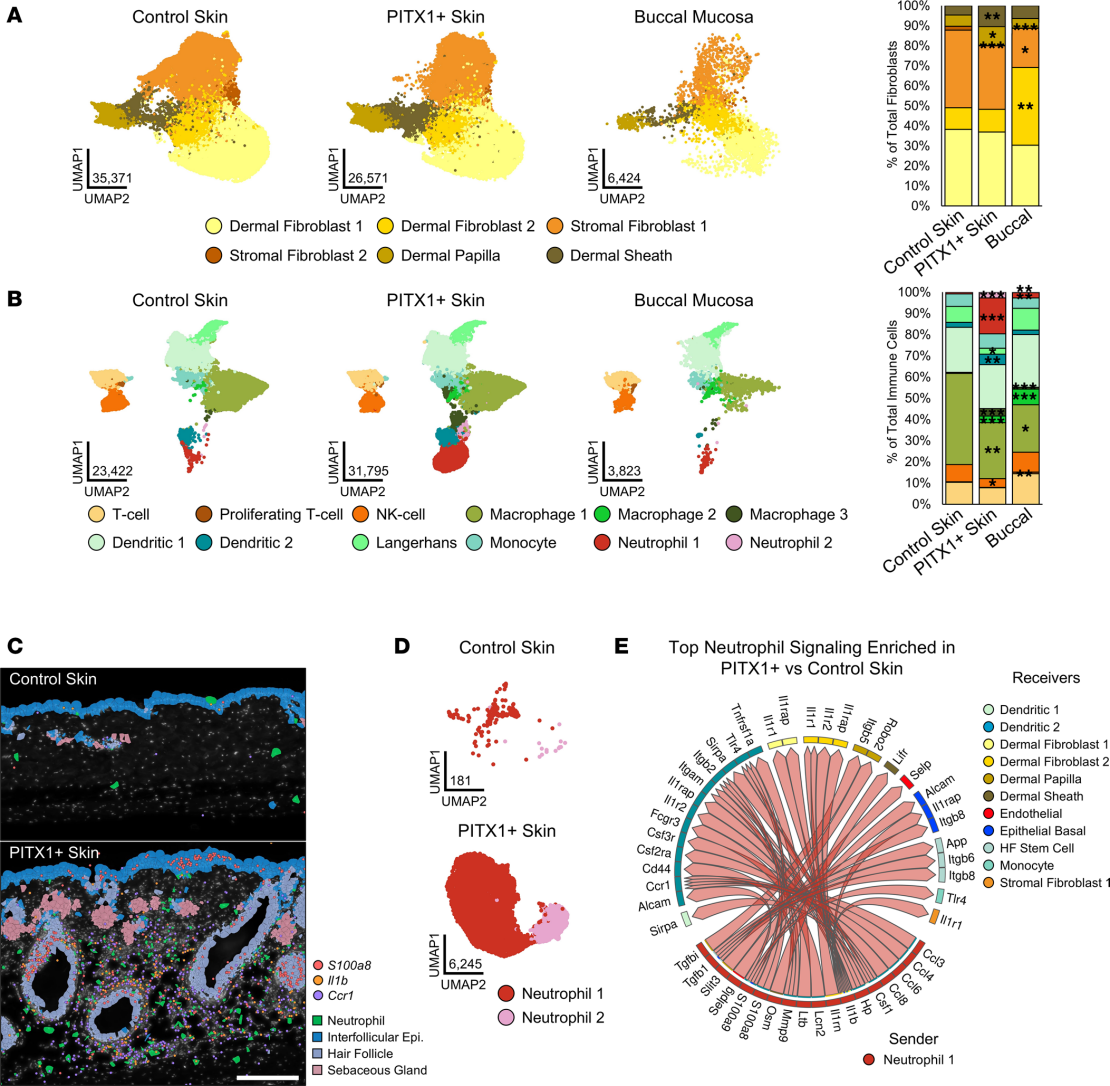
PITX1 expression alters the balance of fibroblasts and immune cells in the skin. (**A**) UMAPs of fibroblast subpopulations in control skin (*n* = 8), PITX1^+^ skin (*n* = 8), and buccal mucosa pools (*n* = 7) (left). Proportion plot of fibroblast subpopulations (right). (**B**) UMAPs of immune cell subpopulations in control skin (*n* = 8), PITX1^+^ skin (*n* = 8), and buccal mucosa pools (*n* = 7) (left). Proportion plot of immune cell subpopulations (right). Significance for proportion plots was assessed by proportionality testing followed by ad hoc comparisons against the corresponding cell type in control skin to derived log_2_ fold-change (log_2_FC) (*P < 0.01 & log_2_FC > |1.5|, **P < 0.01 & log_2_FC > |2|, ***P < 0.01 & log_2_FC > |4|). (**C**) Representative Xenium of male control skin (*n* = 3) and PITX1^+^ skin (*n* = 4), highlighting neutrophils, IFE keratinocytes, HF keratinocytes, and sebaceous gland keratinocytes. Transcripts of *S100a8*, *Il1b*, and *Ccr1* are indicated by colored dots. Scale bar = 200 μm. (**D**) UMAP of control skin and PITX1 skin neutrophil subpopulations. (**E**) Circos plot showing PITX1^+^ skin-enriched ligand-receptor (L-R) interactions predicted by MultiNicheNet to originate from Neutrophil 1 subpopulation (sender). Arrows point to cell subpopulations that are predicted to be engaged by Neutrophil 1 (receivers). Genes constituting predicted signaling pairs are listed on outside of plot.

Six distinct subtypes of fibroblasts were identified from our data set, including 2 populations of dermal (Dermal Fibroblast 1 & 2), 2 populations of stromal/mesenchymal (Stromal Fibroblast 1 & 2), and the fibroblast-like Dermal Papilla and Dermal Sheath cells (Figure 4A). The 2 dermal populations expressed *Col1a1* and *Lum* and were distinguished by an increased expression of inflammatory and dermal papilla/sheath markers within Dermal Fibroblast 2 (*Spon2*, *Cxcl1*, *Tshz3*) (Supplemental Figure 7A). Stromal Fibroblast 1 expressed inflammatory genes (*Cxcl1*, *Cxcl2*, *Plac8*) while Stromal Fibroblast 2 expressed markers of hair follicle–associated and deeper dermal/hypodermal tissue (*Col6a5*). Dermal Papilla (*Bcl2*, *Dkk2*) and Dermal Sheath (*Mylk*, *Tshz3*, *Slit2*) markers were consistent with previous studies on fibroblast heterogeneity with scRNA-Seq (31). Comparatively, buccal mucosa had more Dermal Fibroblast 2 and fewer Stromal Fibroblast 1 & 2 populations than skin (Figure 4A). PITX1-dependent reorganization of the fibroblast led to a decrease in the small population of Stromal Fibroblast 2 cells and an expansion of the Dermal Papilla and Dermal Sheath cells.

### PITX1^+^ skin has a unique balance of immune cells compared with control skin and buccal mucosa.

The buccal mucosa had significantly fewer immune cells compared with control skin while PITX1^+^ skin had a nonsignificant increase (Figure 1C and Figure 4B). The identified subtypes corresponded with lymphoid (T cell, Proliferating T cell, and NK cell) and myeloid (Macrophage 1–3, Dendritic 1 & 2, Langerhans, Monocyte, and Neutrophil 1 & 2) immune populations (Figure 4B and Supplemental Figure 7B). Compared with control skin, the buccal mucosa had proportionally more Macrophage 2 & 3, Neutrophil 1 & 2, and Proliferating T cells and fewer Macrophage 1 cells. The macrophage subtypes shared common myeloid lineage markers *H2-Eb1* and *Cd74* (Supplemental Figure 7B). Macrophage 1 expressed high numbers of *Ccl7*, correlating with previously identified macrophage populations that are immunoregulatory and pro-wound resolution (32, 33). Macrophage 2 was enriched for *Spp1*, a marker of another antiinflammatory macrophage subpopulation (34). Finally, Macrophage 3 cells clustered closely with neutrophils and were enriched with pro-inflammatory markers *Cxcl2*, *S100a8*, and *Cstb*. Both neutrophil populations shared high expression of characteristic markers *Slpi*, *G0s2*, and *S100a8* while the immature neutrophil markers *Ifitm6* and *Retnlg* in Neutrophil 1 distinguished it from Neutrophil 2 (35, 36). PITX1^+^ skin had increased proportions of Macrophage 2 & 3, Dendritic 2, and Neutrophil 1 & 2 populations and decreased proportions of NK cell, Macrophage 1, and Langerhans cells compared with control skin (Figure 4B). Both dendritic subpopulations shared myeloid markers *H2-Eb1* and *Cd74*; Dendritic 2 was distinguished from Dendritic 1 with expression of inflammatory markers *S100a8* and *Cstb*. The remaining immune subpopulations were defined by markers observed in other scRNA-Seq studies: T cell (*Icos*, *Camk4*), Proliferating T cell (*Hist1h1b*, *Top2a*), NK cell (*Trdc*, *Xcl1*), Langerhans (*Cd207*, *Mfge8*), and Monocyte (*Plac8*, *Fn1*) (36–38).

### Other cell type proportions are largely unchanged by PITX1 expression.

Across the tissues, vascular, neural, and muscle cells comprised 5%–15% of single cells captured for sequencing and 10%–40% of cells identified via Xenium (Figure 1C and Supplemental Figure 3D). Buccal samples had salivary gland cells contributing to their total population: approximately 5% in both scRNA-Seq and Xenium data sets. The highly vascularized buccal mucosa is also significantly enriched with vascular cells compared with the skin (5). Endothelial (*Flt1*, *Cd36*, and *Pecam1*) and Lymph Vessel (*Ccl21a*, *Lyve1*, and *Mmrn1*) subpopulations of vascular cells were similar across the samples, with a minor decrease in the proportion of Lymph Vessel cells constituting the PITX1^+^ skin and buccal mucosa (Supplemental Figure 7C). Neural cells were classified as either Myelinating Schwann (*Mpz*, *Ncmap*, *Ctnna3*) or Nonmyelinating Schwann (*Csmd1*, *Kcna2*, *Scn7a*) and Melanocyte (*Mlana*, *Pmel*, *Kit*), pigment-producing, neural crest–derived cells (Supplemental Figure 7D). The proportion of Schwann cells in PITX1^+^ skin and buccal mucosa was no different from control skin, but both tissues had significantly fewer melanocytes. Human oral mucosa contains non-pigment-producing melanocytes at proportions similar to those in our findings (37). Finally, neither the proportions of Vascular Smooth Muscle (*Myh11*, *Tagln*, *Acta2*) nor Skeletal Muscle (*Mylpf*, *Tnnc2*, *Tnni2*) cells significantly changed across the samples (Supplemental Figure 7E). As observed with the keratinocyte subpopulations, the other cell type subpopulations were proportionally shifted toward an oral like state upon cutaneous expression of PITX1^+^, demonstrating a potent keratinocyte-derived paracrine effect on tissue composition and organization.

### Neutrophil influx and signaling is a feature of PITX1-expressing skin.

A significant expansion of neutrophil populations was evident upon PITX1 expression (Figure 4B). Epigenomic evidence points to increased keratinocyte-derived inflammatory factors, such as *Tnf* and *Csf2*, acting as chemoattractants for neutrophils (Supplemental Figure 6B) (38, 39). Furthermore, IPA of differentially expressed genes obtained from bulk RNA-Seq of whole mouse control and PITX1^+^ skin highlights widespread pro-myeloid/granulocyte activation and recruitment pathways (Supplemental Figure 8A). Both Xenium in situ analysis and IHC for the murine neutrophil marker Ly-6G verified the recruitment of neutrophils to PITX1^+^ skin (Figure 4C and Supplemental Figure 8B). These neutrophils tended to cluster around HF-adjacent dermal compartments, with some even invading into the HF bulbs themselves (Supplemental Figure 8B, arrow). Xenium illustrated that PITX1^+^ skin had higher expression of pro-inflammatory genes *S100a8* and *Il1b* in and around the neutrophil-laden areas, with the neutrophils themselves significantly enriched for expression of these markers (Figure 4C and Supplemental Figure 8C). Additionally, a high number of *Ccr1* transcripts, important for neutrophil homing to skin, correlated with areas densely populated with neutrophils (40). Subsetted and reclustered neutrophils from the control and PITX1^+^ skin scRNA-Seq data set retained the original annotations and produced refined markers: Neutrophil 1 (*Retnlg*, *Lcn2*, *S100a8/9*) and Neutrophil 2 (*Cstb*, *Ccl3*, *Mif*, *Atox1*) (Figure 4D and Supplemental Figure 8D). The overwhelming majority (97%) of neutrophils belonged to the Neutrophil 1 population, whose markers suggest predominantly immature or generic inflammatory neutrophils; the remaining percentage (3%) of Neutrophil 2 cells were enriched with immunoregulatory genes. Specifically, Neutrophil 2 gene markers like *Ccl3*, *Mif*, and *Atox1* have been shown to crossactivate other immune cells or are involved with neutrophil recruitment and activation in tissues (41–43). We sought to interrogate how the panoply of immunomodulatory factors expressed by neutrophils in PITX1^+^ skin influences intercellular communication networks. The R package MultiNicheNet can predict intercellular communication in scRNA-Seq of ligands in “sender” cells and receptors in “receiver” cells (44). Using MultiNicheNet, the top sender interactions predicted to originate from Neutrophil 1 cells primarily target other immune cells, fibroblasts, and keratinocytes (Figure 4E). Notably, products of the genes *S100a9*, *Il1b*, and *Tgfb1* were predicted to engage receptors transcribed from genes like *Alcam*, *Il1r1*, and *Itgb8* in dendritic cells, dermal fibroblasts, and dermal adjacent keratinocyte subpopulations. These ligand-receptor pairs have been reported to moderate inflammation within the context of cancer, inflammatory conditions, and wound healing (45–47). Overall, PITX1^+^ skin increases the quantity of actively signaling neutrophils that may be key actors in reshaping tissue morphology.

### PITX1^+^ skin is dominated by an immune cell signaling signature and acquires buccal intercellular communication networks.

**Figure 5:**
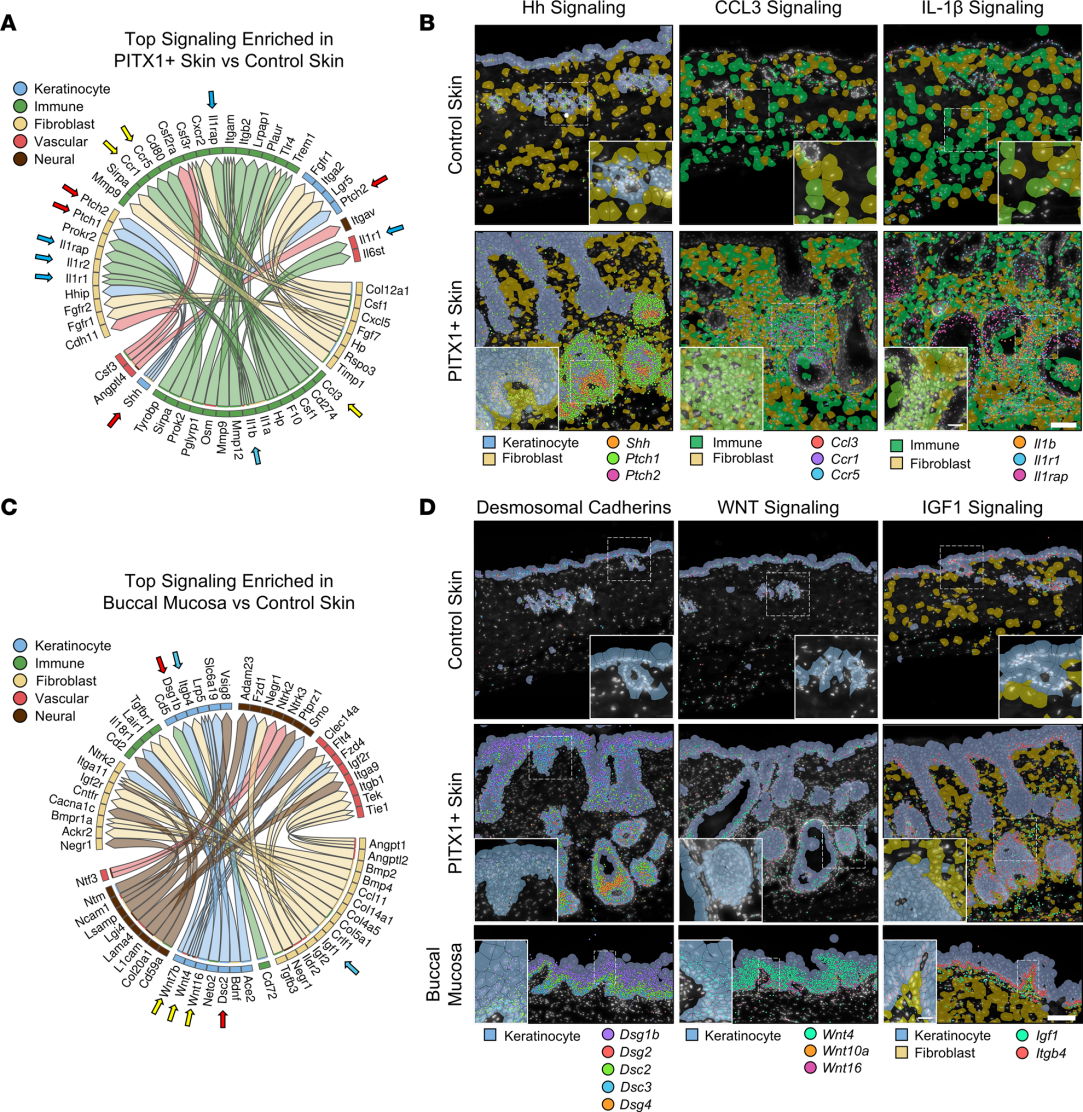
Intercellular communication in the skin is shifted toward an oral like state by PITX1. (**A**) Circos plot showing the top 20 PITX1^+^ skin-enriched L-R interactions predicted by MultiNIcheNet between cell types of the skin. Control (*n* = 8) and PITX1^+^ (*n* = 8). Genes constituting predicted signaling pairs are listed on outside of plot. (**B**) Representative Xenium of male control skin (*n* = 3) and PITX1^+^ skin (*n* = 4) of indicated L-R pairs (arrows in **A**) highlighting Hedgehog (Hh), CCL3, and IL-1β signaling. Dashed boxes show inset area. Transcripts indicated are represented by colored dots. Scale bar = 100 μm; inset scale bar = 25 μm. (**C**) Circos plot showing the top 20 buccal mucosa–enriched L-R interactions over control skin predicted by MultiNIcheNet. Buccal mucosa (*n* = 7). (**D**) Representative Xenium of male control skin, PITX1^+^ skin, and buccal mucosa (*N* = 7) of indicated L-R pairs (arrows in **C**) highlighting expression of desmosomal cadherins and WNT and IGF1 signaling pathways. Dashed boxes show inset area. Transcripts indicated are represented by colored dots. Scale bar = 100 μm; inset scale bar = 25 μm.

The top differentially predicted ligand-receptor pairings across all cell types in the PITX1^+^ skin were signaling networks predominantly arising from immune cells and were verified by Xenium (Figure 5, A and B, and Supplemental Figure 9). Interestingly, the only predicted factor originating from keratinocytes was *Shh*, the prototypical Hedgehog (Hh) signaling pathway ligand. SHH binds the receptor PTCH1 (*Ptch1*) and, to a lesser extent, PTCH2 (*Ptch2*) to engage downstream cellular signaling. Hh signaling is critical to de novo hair follicle morphogenesis, epidermal homeostasis, and hair follicle cycling (48). Here, *Shh* is predicted to engage both keratinocyte and fibroblast *Ptch1* and *Ptch2* receptors (Figure 5A). We observed that *Shh* transcripts were largely expressed in the HF matrix while *Ptch1* and *Ptch2* are expressed in the same cells and in dermal sheath and dermal papilla cells surrounding the hair bulb (Figure 5B and Supplemental Figure 10A). SHH/PTCH1 signaling between HF matrix and dermal papilla cells is known to influence dermal papilla maturation and maintenance of the HF cycle. *Ccl3* originating from immune cells was predicted to bind *Ccr1* and *Ccr5* in most immune cells and a subpopulation of fibroblasts (Figure 5A and Supplemental Figure 10A). The CCL3-CCR1/5 signaling axis is known to promote the recruitment of granulocytes, including neutrophils, to the skin (49). Xenium data illustrated high concentrations of these transcripts in regions of PITX1^+^ skin that are densely packed with immune cells, suggesting a self-sustaining pro-inflammatory reaction is occurring. Another chemoattractant and immunoactivating ligand, *Il1b*, is predicted to originate from the immune cells, mainly neutrophils, and interact with its cognate receptors *Il1r1*, *Il1r2*, and *Ilrap* in fibroblasts, vascular cells, and other immune cells (Figure 5A and Supplemental Figure 10A). Like *Ccl3*, these factors appear to be highly concentrated in the densely packed immune fibroblast niches surrounding the hair follicles of PITX1^+^ skin (Figure 5B). In addition to a pro-inflammatory role, IL-1β stimulates fibroblast proliferation, migration, and matrix remodeling functionality (50). Importantly, it does not seem to promote the conversion of fibroblasts to myofibroblasts and may actively antagonize that alteration. Thus, there is a fine balance of immunomodulatory and pro- and antifibrotic signaling networks co-occurring in the PITX1^+^ skin.

PITX1^+^ drastically reshapes the morphology and molecular networks of the skin, but what crucial signaling networks are influencing mucosal biology compared with healthy skin is not well characterized. Our buccal mucosa scRNA-Seq data suggested that the most differentially active intercellular signaling networks span all the cell types (Figure 5C). We predicted that many of these buccal predominant signaling pairs co-occur in the PITX1^+^ skin. Indeed, PITX1^+^ skin expressed a complement of desmosomal cadherins akin to buccal mucosa and had increased gene expression of signaling molecules that constitute WNT and IGF1 pathways (Figure 5D). Desmosomal cadherins are spatiotemporally expressed during keratinocyte differentiation and are known to modulate cellular signaling networks in a non-adhesion-dependent manner (51). Desmosomal cadherin organization throughout the IFE keratinocytes was similar among control and PITX1^+^ skin and oral mucosa (Figure 5D and Supplemental Figure 10B) (52, 53). Notably, there was a significant increase in the expression of basal/activated keratinocyte cadherins *Dsg2* and *Dsc2* in PITX1^+^ skin. WNT signaling is another key cell signaling pathway that is important for epidermal homeostasis, maintenance, and hair follicle renewal (54). While no cognate receptors for Wnts were included in the Xenium panel, buccal mucosa had much higher expression of Wnt family genes (Figure 5D). Specifically, *Wnt10a* and *Wnt16* were expressed in basal oral keratinocytes while *Wnt4* was expressed upon differentiation (Supplemental Figure 10B). PITX1^+^ keratinocytes had a moderate increase in Wnt gene expression. The hemidesmosomal β4-integrin (*Itgb4*) is expressed in both oral and cutaneous keratinocytes at the DEJ, is critical for barrier integrity, and can be engaged by IGF1 to promote anchorage-independent growth (55). A high concentration of fibroblast-derived *Igf1* transcripts were proximal to basal keratinocyte-derived *Itgb4* in the buccal mucosa (Figure 5, C and D, and Supplemental Figure 10D). While both control and PITX1^+^ keratinocytes expressed *Itgb4* at the DEJ, there was a significant increase in *Igf1* transcripts in the fibroblasts of the PITX1^+^ skin. These data suggest that PITX1 is not only driving a shift in the transcriptional programming of epidermal keratinocytes toward an oral keratinocyte signature but also influencing broader tissue signaling networks and remodeling of local cell-cell interactions toward an oral like state.

### PITX1 promotes cutaneous wound healing.

**Figure 6:**
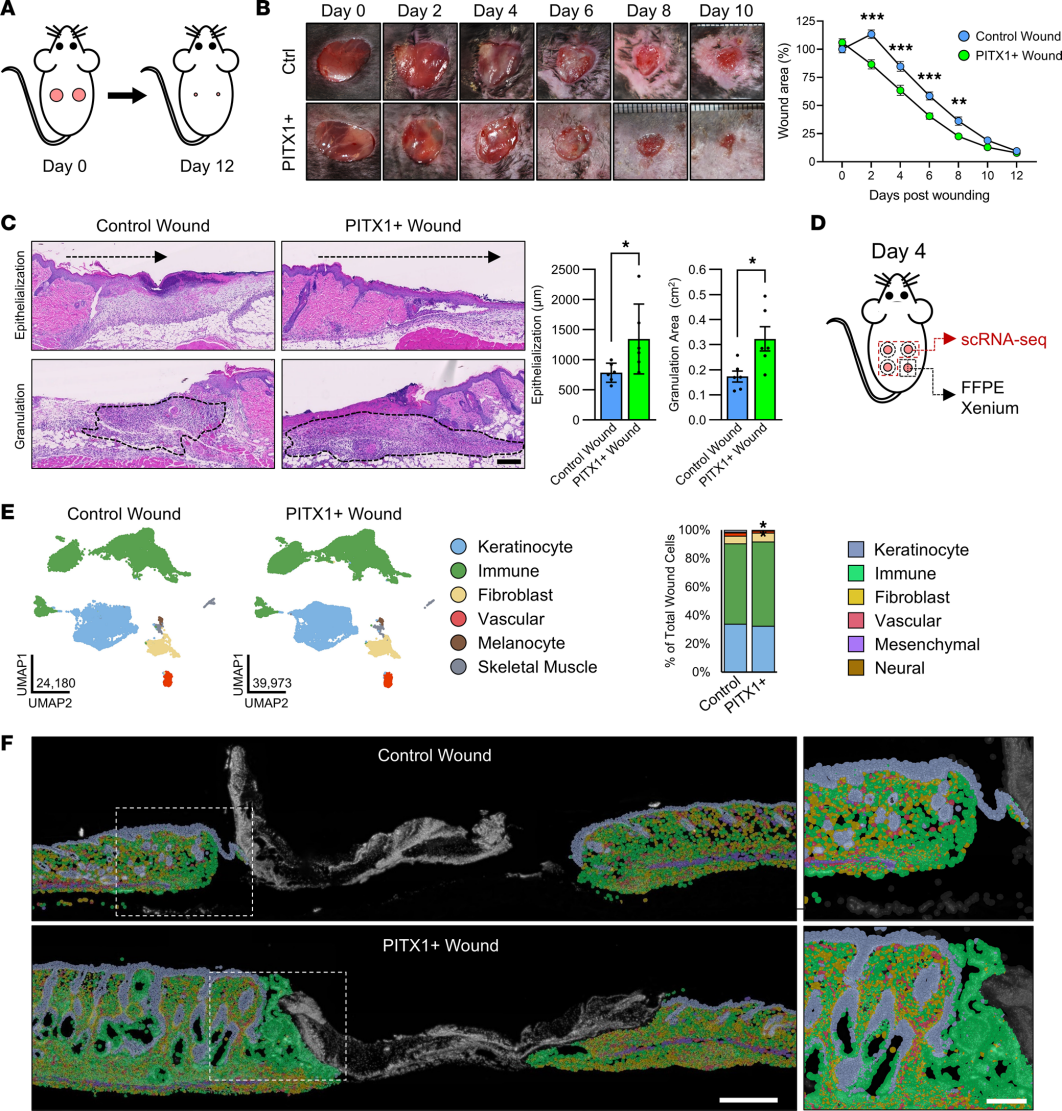
PITX1 promotes cutaneous wound healing. (**A**) Schematic of full-thickness wound-healing model. (**B**) Representative images from control (*n* = 11) and PITX1^+^ (*n* = 11) mice upon wounding (day 0) until day 10 after wounding (left). Quantification of wound area remaining open during healing. Significance assessed by 2-way repeated measures ANOVA with Tukey’s post hoc testing (**P < 0.01, ***P < 0.001). (**C**) Representative H&E images of male day 4 wounds used for measurement of wound reepithelialization and area of granulation tissue (left). Scale bar = 100 μm. Quantification of the reepithelization distance and granulation tissue area plotted as average ± SEM (right). Significance assessed by Student’s 2-tailed *t* test (*P < 0.05). (**D**) Schematic of wound tissues used for scRNA-Seq and Xenium. (**E**) UMAPs and cell type annotations of control (*n* = 6) and PITX1^+^ (*n* = 7) skin wound scRNA-Seq (left) and proportion plot of cell types (right). Significance for proportion plot assessed by proportionality testing followed by ad hoc comparisons against the corresponding cell type in control wound skin to derive log_2_ fold-change (log_2_FC) (*P < 0.01 & log_2_FC > |1.5|). (**F**) Xenium in situ representative images of FFPE male control (*n* = 1) and PITX1^+^ (*n* = 2) wound sections with cell types highlighted. Dashed box highlights wound-adjacent inset image. Scale bar for whole wound = 500 μm, scale bar for wound-adjacent inset = 200 μm.

Oral wound healing is characterized by rapid closure and superior resolution of the injury compared with skin (4, 5). Our data suggested that PITX1^+^ keratinocytes are more proliferative, are more migratory, and express an oral like transcriptional network and that resident cell populations of the skin are subsequently adapting. To test these effects on wound healing dynamics, we performed full-thickness excisional wounding with 6 mm biopsies on the dorsa of control and PITX1^+^ mice and tracked healing over 12 days (Figure 6A). PITX1^+^ mice were found to heal at a rate faster than control during the initial stages (day 2–8) of injury repair as measured by the percentage of the wound remaining open compared with day 0 (Figure 6B). This timeline corresponds with the inflammatory and proliferative phases of wound repair (1). By day 4 after wounding, both the area of granulation tissue and the distance traveled by the nearby IFE into the wound were significantly greater in the PITX1^+^ mice (Figure 6C). Control and PITX1^+^ wounds were fully closed within 2 weeks and were undergoing similar tissue remodeling 35 days after injury (Supplemental Figure 11A). We did not observe any changes in the organization or integrity of the extracellular matrix in PITX1^+^ mice, suggesting that wound healing in these mice is not accompanied by increased fibrosis.

We performed both scRNA-Seq and Xenium in situ on the control and PITX1^+^ wounds. scRNA-Seq studies of cutaneous wounds have identified a myriad of novel insights (17, 23, 31, 56). Four 3.5 mm, full-thickness wounds were inflicted on the dorsum of control and PITX1^+^ mice. On day 4 after wounding, 3 of these wounds were excised by centering 6 mm biopsies over the partially healed wounds and subsequently dissociated for scRNA-Seq. The remaining wound was dissected from the dorsal skin, bifurcated at the wound midline, and fixed and embedded for Xenium analysis (Figure 6D). Following data processing and quality control, a total of 64,153 cells (24,180 control wound cells and 39,973 PITX1^+^ wound cells) from control (*n* = 6) and PITX1^+^ (*n* = 7) wounds were annotated (Figure 6E and Supplemental Figure 11, B–E). The proportions of cells captured from control and PITX1^+^ wounds were relatively equal: keratinocytes (33.8%/32.3%; control/PITX1^+^), immune cells (56.5%/59.2%), fibroblasts (5.4%/6.3%), vascular cells (2.2%/1.4%), melanocytes (0.4%/0.4%), and skeletal muscle cells (1.7%/0.4%). Only vascular and skeletal muscle cells had significant, but minor, decreases upon PITX1^+^ induction (Figure 6E). Xenium analysis of the FFPE wounds captured similar proportions of cell populations (Figure 6F and Supplemental Figure 12). The eschar contained mostly dead or dying cells and was excluded from Xenium analysis. A total of 110,159 wound and wound-adjacent cells (13,777 control and 96,382 PITX1^+^ cells) were analyzed by Xenium, and, like the healthy tissues, had proportional differences in the cellular composition when compared with its corresponding scRNA-Seq data set: keratinocytes (37.8%/26.2% control/PITX1^+^), immune cells (29.5%/44.4%), fibroblasts (12.2%/20.1%), vascular cells (5.3%/4.3%), neural cells (1.3%/1/.1%), and mesenchymal cells (7.0%/3.9%; majority were skeletal muscle) (Supplemental Figure 12, B–C). There were no differences in the proportions of the cell types between control and PITX1^+^ wounds in the Xenium data. Though significance could not be assessed because of the low number of samples, there was increased cellular density in the PITX1^+^ wound skin (Supplemental Figure 12D).

### PITX1^+^ wounds have a preactivated epithelium and increased neutrophil recruitment.

**Figure 7:**
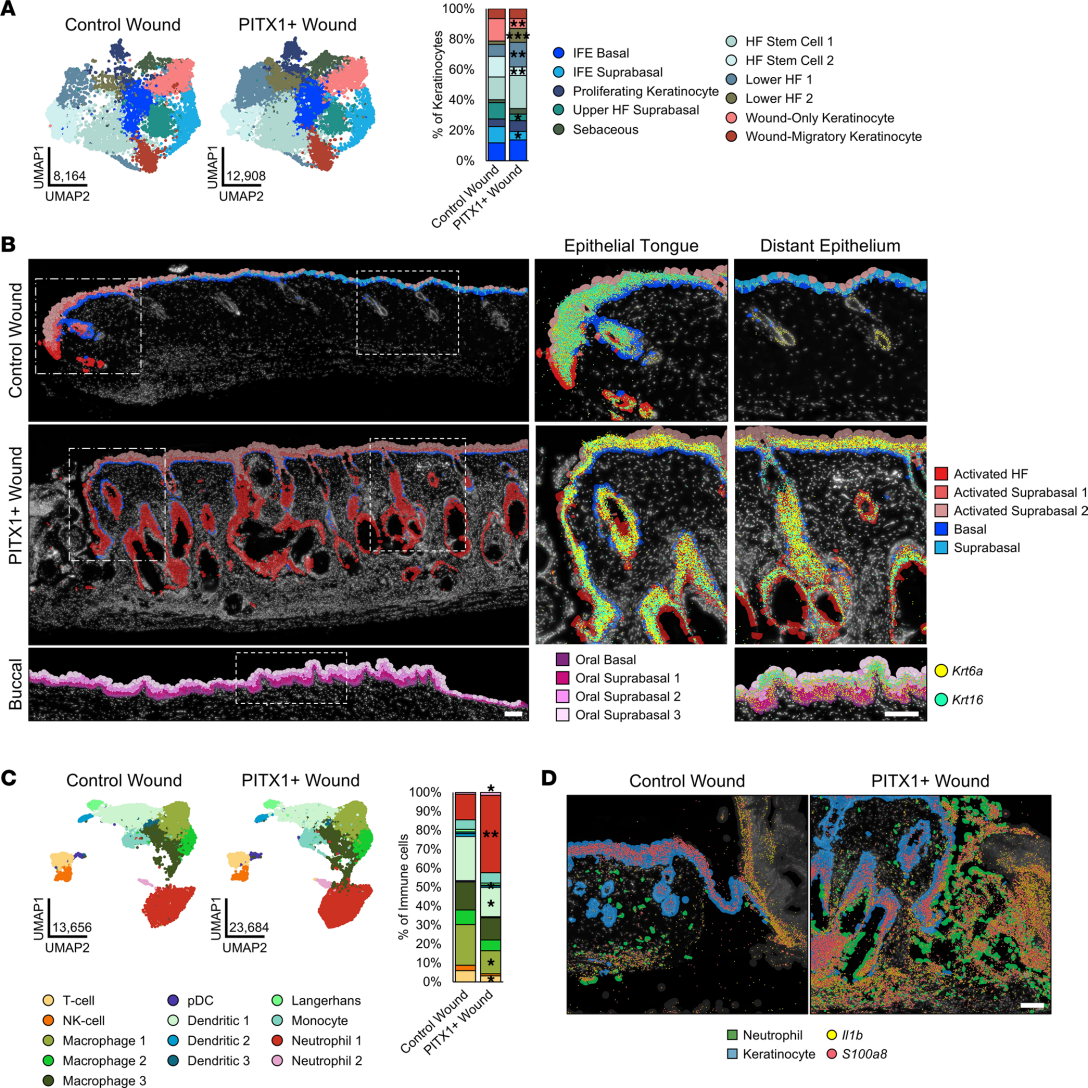
Primed, activated keratinocytes and enhanced neutrophil recruitment are features of PITX1^+^ skin wounds. (**A**) UMAPs of control skin (*n* = 8), PITX1^+^ (*n* = 8), and buccal mucosa (*n* = 7) scRNA-Seq keratinocyte subpopulations (left). Proportion plot of wound keratinocyte subpopulations (right). (**B**) Representative Xenium of male control (*n* = 1) and PITX1^+^ wounds (*n* = 2) with cell subtypes highlighted. Dotted boxes indicate magnification of the wound-adjacent epithelial tongue (middle) and distal epithelium (right). *Krt6a* (yellow) and *Krt16* (cyan) transcripts represented by colored dots. Scale bars of both large wound field and insets are 100 μm. (**C**) UMAPs of control and PITX1^+^ wound scRNA-Seq immune cell subpopulations (left). Proportion plot of immune cell subpopulations (right). pDC, plasmacytoid dendritic cell. (**D**) Representative Xenium of wound-adjacent region of male control and PITX1^+^ samples with Neutrophils (green) and Keratinocytes (blue) highlighted. *Il1b* (yellow) and *S100a8* (red) transcripts represented by colored dots. Scale bar = 100 μm. Significance for proportion plots was assessed by proportionality testing followed by ad hoc comparisons against the corresponding cell type in control skin to derive log_2_ fold-change (log_2_FC) (*P < 0.01 & log_2_FC > |1.5|, **P < 0.01 & log_2_FC > |2|, ***P < 0.01 & log_2_FC > |4|).

The keratinocytes that comprise the wound-adjacent and proximal epithelium largely recapitulate the scRNA-Seq subtypes observed in healthy tissue (Figure 7A). A smaller proportion of HF keratinocytes, compared with the healthy skin scRNA-Seq data set, was observed in both conditions (51.2% control wound, 60.7% PITX1^+^ wound versus 72%/75% in healthy tissue) (Supplemental Figure 4C). Many of the same markers defining the wound keratinocyte subtypes were shared with the healthy skin subtypes (Supplemental Figure 4B and Supplemental Figure 13A). There were proportionally fewer IFE Suprabasal (*Krt10*, *Serpinb3b*), Upper HF Suprabasal (*Cst6*, *Defb6*), and HF Stem Cell 2 (*Cd34*, *Tgm5*) cells and more Lower HF 1 & 2 (*Sox5*, *Ptch1*; *Lef*, *Pik3r3*, respectively) PITX1^+^ keratinocytes (Figure 7A). Wound-Migratory and Wound-Only keratinocytes were unique subtypes to the wound scRNA-Seq data set. Wound-Migratory keratinocytes (*Igfbp3*, *Itga2*) are those that are at the leading edge of the migrating epithelial tongue and consist of a population of highly mobile, partially dedifferentiated, and semiproliferative cells (57). Wound-Only keratinocytes (*Stfa1*, *Sprr2a3*) are the keratinocytes distal to the leading edge and have been shown to proliferate and differentiate as healing progresses (58). While both control and PITX1^+^ wounds had proportionally equivalent numbers of Wound-Migratory cells, PITX1^+^ had significantly fewer Wound-Only keratinocytes (Figure 7A). Xenium analysis of the wounds revealed that virtually all the keratinocytes of the PITX1^+^ IFE and hair follicles are activated (high expression of *Krt6a* and *Krt16*) (Figure 7B). The Xenium keratinocyte clusters Activated HF, Activated Suprabasal 1, and Activated Suprabasal 2 expressed genes involved in wound-responsive keratinocyte activation, such as keratins (*Krt6a/b*, *Krt16*), inflammation-related (*S100a8*, *Il1b*, *Il1rn*, *Slpi*), and basal/oral like keratinocyte markers (*Dsc2*, *Aldh1a3*, *Krt4*, *Tgm3*) (Supplemental Figure 13D) (26). Healthy oral keratinocytes constitutively expressed some of these markers, including *Krt6a* and *Krt16*, in the buccal mucosa, supporting prior observations of them existing in a primed and activated state compared with epidermal keratinocytes (Figure 7B) (4). Indeed, the Xenium Activated Suprabasal 1 & 2 clusters were present in the expected niche of scRNA-Seq Wound-Only cells — the distal portion of the wound tongue. No Activated HF and significantly fewer Activated Suprabasal 1 and 2 cells were found in control epidermis distant from the wound edge, its unwounded IFE being constituted by populations of healthy Basal (*Col17a1*, *Hist1h1b*) and Suprabasal (*Krt77*, *Egfr*) keratinocytes. Together, PITX1^+^ keratinocytes exist in an activated state (Krt6a^+^, Krt16^+^, Aldh1a3^+^, S100a8^+^) in the healthy epidermis, priming them for a rapid response to tissue injury through increased proliferation and migration.

Immune cell subtypes were significantly changed with PITX1 expression in the wounds. T cell (*Icos*, *St6galnca3*), NK cell (*Trdc*, *Xcl1*), Macrophage 1 (*Ccl8*, *Cbr2*), and Dendritic 1–3 (*H2*-*Ab1*, *Tmem176b*; *Cacnb3*, *Ccl22*; *Cxcl10*, *Ifit1*, respectively) proportionally decreased while Neutrophil 1 & 2 (*Retnlg*, *G0s2*; *S100a8*, *Ccl4*, respectively) increased (Figure 7C and Supplemental Figure 13B). Ly-6G IHC verified the increase in neutrophils recruited to the PITX1^+^ wound region (Supplemental Figure 13C). Most neutrophils belonged to the Neutrophil 1 subtype versus Neutrophil 2 (93.5% and 96.3% in control and PITX1^+^, respectively), and Neutrophil 2 was only distinguished from Neutrophil 1 by its high expression of the chemokine *Ccl4* (Supplemental Figure 13B). Xenium showed that these wound-responding neutrophils release increased amounts of *Il1b* and *S100a8* into the wound bed of PITX1^+^ mice (Figure 7D). Importantly, *Il1b* and *S100a8* were detected in both the wound-adjacent skin as well as the nearby eschar. Indeed, MultiNicheNet predicted increased intercellular communication originating from these 2 scRNA-Seq neutrophil populations in the PITX1^+^ wounds (Supplemental Figure 13E). They were predicted to engage fibroblast, immune, vascular, and keratinocyte populations via inflammatory molecules, such as *Tnf*, *Spp1*, and *Mmp9*, all key factors influencing cellular response to wounding (34, 38, 59). Ectopic PITX1 expression in epidermal keratinocytes partially reprogrammed them to an oral like state, simultaneously activating the keratinocytes and promoting downstream modulation of the cellular milieu of the skin. Ultimately, these induce drastic morphologic alterations in the skin and promote wound healing via increased reepithelialization and pro-resolution immunoregulatory interactions.

## Discussion

This study demonstrates that PITX1 is a powerful driver of an oral keratinocyte transcriptional program that has pleiotropic effects on keratinocyte lineage and interactions with other tissue-resident cells and on overall tissue morphology. We show that ectopic expression of PITX1 drives alopecia in the murine skin by irreversibly halting the hair cycle and promoting the terminal differentiation of keratinocyte subpopulations that drive hair follicle renewal. PITX1^+^ and oral keratinocytes are more proliferative and migratory than control keratinocytes. We and others have shown that oral keratinocytes (murine or human) proliferate and migrate faster than cutaneous keratinocytes, possibly contributing to their enhanced ability to reepithelialize wounds (3, 4). Furthermore, knockdown of PITX1 in oral keratinocytes impairs their migration through negative transcriptomic regulation of migration-associated genes while transduction of PITX1 into epidermal keratinocytes conversely confers pro-migratory potential to the cells. Both the skin and oral mucosa undergo similar processes of terminal differentiation, but buccal keratinocytes do not keratinize like the skin and exist in an environment kept constantly moist and unexposed to UV damage (60).

Newer single-cell and spatial transcriptomic techniques are revolutionizing our understanding of the precise molecular interactions that support tissue development. Here, we leverage these techniques to disentangle the effects of PITX1 expression in the skin and how that compares with buccal mucosa. Both techniques allow identification of the tissue-constituent cell types based on transcriptome gene markers and have allowed us to demonstrate that epidermal keratinocytes expressing PITX1 acquire an oral like state. PITX^+^ epidermal keratinocytes express oral genes during differentiation and acquire global transcriptomic features of oral keratinocyte subpopulations. This has the morphologic consequence of inducing epidermal hyperplasia and hair follicle abnormalities. In effect, the transcriptomic conversion disrupts the hair follicle cycle program, leading to alopecia through a combination of proliferation of the paused anagen keratinocytes, terminal differentiation in the hair shafts, and breakdown of the immunologic privilege of the hair follicle, leading to loss of the hair follicle stem cells (HFSCs). Perturbation of hair follicle niche viability is a feature of mammalian aging, leading to HFSC quiescence and hair loss (61).

A lineage-defining role of PITX1 has recently been shown in corneal epithelium as well. A RORA/PITX1 signaling axis underpins the specific differentiation of limbal stem/progenitor cells to corneal epithelium cells and suggests a role for RA signaling relating to PITX1 (13). We identified the retinaldehyde dehydrogenase isoform *ALDH3A1* as an oral signature gene (4). The mouse skin-specific isoform and epidermal stem cell marker, *Aldh1a3*, was upregulated upon PITX1 expression and was also a marker of activated hair follicles in wounded skin (62). Both *ALDH3A1* and *Aldh1a3* facilitate the critical step of converting retinal to bioactive RA within cells (63). RA biosynthesis and signaling are critical for the process of wound-induced hair neogenesis, a unique feature of large (>1 cm) murine wounds to consistently regenerate hair follicles in the center of the wounds (64, 65). All-trans RA (atRA) is a key mediator of HFSC lineage plasticity (66). Importantly, temporal control of exogenous atRA within the hair follicle niche is required for HFSCs to participate in wound healing. Withdrawal of either atRA or its precursor, vitamin A, potentiates HFSC migration to the wound bed but prevents HF neogenesis in the healed skin. PITX1^+^ skin and oral mucosa both prominently expressed the intracellular RA nuclear shuttle *Crabp2* during keratinocyte differentiation, suggesting an increased flux of atRA or other RA isomers in these tissues. The link between PITX1 and RA signaling is unclear, and how RA-related signaling may distinguish oral and cutaneous keratinocyte stem cell dynamics requires further study. The sebaceous glands of the mice are hypermorphic and, curiously, do not seem to have increased proliferation. Sebaceous glands have *Krt5* expression in a pool of self-contained sebocyte stem cells that participate in wound reepithelialization (67). What contextual signaling occurs in the sebaceous and in the presence of PITX1 that does not lead them to proliferate or terminally differentiate is unknown.

CUT&Tag-Seq efficiently defines genomic occupancy of histones and transcription factors in far fewer cells and in more hospitable conditions than ChIP-Seq (28). CUT&Tag-Seq for PITX1 allowed us to reliably discern binding adjacent to genes that had increased expression following expression of PITX1 in the skin. PITX1 expression did not influence the expression of the other oral signature genes (*Pitx2*, *Sox2*, *Pax9*) or *Trp63*, a key regulator of epidermal development and differentiation (68). Activated keratinocytes are defined, in part, by expression of keratins, such as *Krt6a/b*, *Krt16*, and *Krt17*, and are the wound-responding keratinocytes that quickly migrate into the site of injury, proliferate, and differentiate into the epithelial barrier. Though activated keratinocytes play a key role in responding to injury, chronically activated keratinocytes may contribute to nonhealing wounds in patients with diabetes (69, 70). Oral keratinocytes are not activated per se but are primed for a wound response by tighter regulation of inflammatory and other damage-associated genes (4). Our epigenomic data suggest PITX1 is a key player in maintaining a balance between activation and priming within keratinocytes. This activation ultimately leads to a PITX1-dependent potentiation of keratinocyte migration and proliferation in vitro and in vivo (Supplemental Figure 1, C and D). Furthermore, PITX1 has been shown to be overexpressed in cutaneous SCC and regulate the function of tumor-propagating cells (12). We did not observe spontaneous tumorigenesis in either healthy or wounded PITX1^+^ skin in the limited time scale we conducted these studies, suggesting that PITX1 expression alone or in combination with wounding is not sufficient for immediate tumor initiation. While PITX1 itself binds classically activated keratinocyte marker genes and promotes global activation of pathways related to wound healing and epithelialization, indirect transcriptomic alterations, evidenced by the set of genes with increased H3K4me3 occupancy at their promoters, also suggests that the keratinocytes express products that promote inflammation. Increased H3K4me3 occupancy at pro-inflammatory genes such as *Tnf* and *Csf2* and increased expression of inflammation genes such as *S100a8* suggests PITX1^+^ primes the skin for an inflammatory response.

While PITX1^+^ skin had a statistically nonsignificant increased proportion of immune cells compared with the control skin, the distribution of immune cell subtypes, particularly neutrophils, was apparent. Prior scRNA-Seq studies have established that the census of immune cells drastically varies in different oral and cutaneous sites (37, 56, 71). Notably, neither healthy buccal mucosa nor skin has significant populations of tissue-resident neutrophils. Neutrophils are recruited to the skin following injury to deposit proinflammatory, antimicrobial, and tissue-remolding granules. The influx of neutrophils into PITX1^+^ skin creates a pro-inflammatory environment in the tissue, evidenced by the increased number of *S100a8* and *Il1b* transcripts. These neutrophils may home to the skin through the CCL3-CCR1/5 signaling axis, though future work will be necessary to elucidate the precise mechanism (49). Additionally, these neutrophils are predicted by MultiNicheNet to engage a variety of neighboring cells across cell types. Indeed, the highest source of predicted cell-cell interactions in the PITX1^+^ skin compared with control skin are from immune cells, with a bulk of that signaling emanating from neutrophils themselves. Identification of reliable transcriptomic markers to discern neutrophil heterogeneity is still forthcoming and will help distinguish if these neutrophils are pro- or antiinflammatory (72).

PITX1 promoted wound healing in the skin by increasing cellular migration of keratinocytes and stimulating granulation tissue formation. The combination of scRNA-Seq and Xenium data sets allows more finite analysis of the wound-healing process. PITX1 expression induced keratinocyte activation in both healthy and wound-adjacent epidermis (evidenced by *Krt6a*, *Krt16*, *S100a8*, *Dsc2*, and *Aldh1a3* expression). Keratinocyte activation is classically defined by expression of markers such as *Krt6a* and *Krt16* and delineates a pro-migratory and pro-proliferative phenotype that is ordinarily initiated during the wound healing process or disease (26). Importantly, spatiotemporal regulation typically limits keratinocyte activation to only the cells migrating into or immediately adjacent to the wound bed. Given the observed nearly constant activation of PITX1^+^ keratinocytes, they are primed to rapidly respond to tissue injury by migrating and proliferating into the wound bed. Like healthy tissue, PITX1^+^ wounds are characterized by a significant influx of neutrophils to the wound site. They are required for successive healing as abrogated recruitment of neutrophils to wounds is a feature of chronic, nonhealing diabetic foot ulcers (DFUs) (73). Wound-responsive neutrophils have also been shown to release condensed chromatin decorated with antimicrobial proteins (neutrophil extracellular traps, or NETs) during NETosis (74). NETs impair healing in the skin and are extensively found in DFUs and other chronic wounds (75). NETosis occurrence is low in both healthy wounds and DFUs that eventually heal by the recruitment and activation of FOXM1^+^ neutrophils while nonhealing DFUs have low expression of *FOXM1*. We did not determine if persistent recruitment of neutrophils to PITX1^+^ skin stimulates damaging NETosis to occur.

Together, our study provides evidence that ectopic PITX1 is a driver of oral keratinocyte identity and tissue dynamics that enhances wound healing in the skin. Clinical efforts to improve cutaneous wound healing have largely revolved around improving existing bandaging or skin-grafting technologies, but development of targeted therapeutics that potentiate the natural healing process has been stagnant (76). This work illustrates that modulating the tissue identity of the epithelium is sufficient to drive tissue-wide reorganization to promote healing. The resources produced by this study, including the scRNA-Seq data, Xenium in situ data, and CUT&Tag-Seq data sets, will be helpful for future studies and serve as a model of how these newer technologies and comparative studies may be combined to glean deeper insights into biological processes. Importantly, capturing tissue-wide transcriptomic dynamics will be vital to acquiring a thorough understanding of processes, such as wound healing, and potentially allow for the development of more efficacious therapeutic modalities for both acute and chronic, nonhealing wounds.

## Methods

### Sex as a biological variable.

Our study involved the use of both male and female mice and found equivalent results between both sexes.

### Statistics.

Explanation of statistics can be found in Supplemental Methods.

### Study approval.

The NIH Institutional Animal Care and Use Committee has approved the mouse studies conducted for this work under protocol number A022-01-01.

### Data availability.

All data used for the generation of graphs and figures are available in the Supporting Data Values. Datasets for scRNA-Seq, RNA-Seq, Xenium in situ, and CUT&Tag-Seq have been deposited in NCBI GEO database, accession GSE280088. R code that was used to process, analyze, and present scRNA-Seq and Xenium data was deposited to GitHub repository overmillera/pitx1-2024-jci-insight (https://github.com/overmillera/pitx1-2024-jci-insight; commit ID ab0222476d335ddc7099c1e7f964521e8b94ed46).

## Author contributions

Conceptualization was performed by AMO, AU, and MIM. Experimental methodologies were conceived by AMO, AU, and MIM. Experiments were performed by AMO, AU, EDH, CGO, KH, and FN. Mouse studies were conducted by AMO, AU, EDH, and KH. Genomic data collection and processing were performed by AMO, AU, EDH, FN, SD, SRB, and KJ. Analysis of experimental data was performed by AMO, AU, EDH, SN, SRB, KJ, and MIM. Writing of the manuscript draft and figure organization were performed by AMO and MIM. Review and editing of the manuscript were performed by AMO, SN, CGO, KH, SD, SRB, KJ, and MIM. Resources were contributed by YWC and SD. Supervision and funding were contributed by MIM. All authors read and approved the manuscript prior to submission.

## Supplementary Material

Supplemental data

Supplemental table 1

Supporting data values

## Version 1 10/31/2024

In-Press Preview

## Version 2 12/20/2024

Electronic publication
