# Supplementary material for: Reprogramming of epidermal keratinocytes by PITX1 transforms the cutaneous cellular landscape and promotes wound healing
Source: JCI Insight. 2024 Dec 20;9(24):e182844. doi: 10.1172/jci.insight.182844 (PMC11665584; doi:10.1172/jci.insight.182844)
Supplement: Supplemental data [file jciinsight-9-182844-s158.pdf]

SUPPLEMENTAL MATERIAL

METHODS AND MATERIALS

Generation of PITX1 transgenic mice

TRE-*Pitx1* mice provided by Dr. Yi-Wen Chen (1) were crossed with *Krt5*-rtTA mice (Jackson Laboratory; #017519) to generate a doxycycline-inducible epithelial-specific PITX1 transgenic mice (TRE-*Pitx1*/*Krt5*-rtTA). Adult P56-70 (second telogen) TRE-*Pitx1*/*Krt5*-rtTA mice were fed doxycycline chow (200mg/kg daily) for 6 weeks to induce PITX1 expression and allow for a complete turnover of the epidermis prior to experiments; littermates were fed normal chow as controls. Both sexes of mice were used in the control and PITX1+ groups, zygosity of either the *Krt5* or *Pitx1* transgenes was found not to alter experimental results, and doxycycline fed to wild-type (*Krt5*-rtTA) mice was found to not alter baseline characteristics of the skin or the wound healing response (data not shown).

Mouse tissue harvesting

Following 6 weeks of normal or doxycycline feed (P98-112), mice were euthanized by CO_2 asphyxiation and cervical dislocation per NIH IACUC guidelines. For skin, the mouse dorsum was shaven and wiped clean of loose hairs with 70% ethanol. Shaved areas were depilated with application of Nair™ for 3 minutes prior to swabbing and rinsing with cosmetic cotton applicator pads dipped in ultrapure water (KD Medical; #RGF3410) and another wipe of 70% ethanol. Skin was dissected from the mouse and extraneous subcutaneous fat removed by scalpel. Buccal mucosa was removed by completely bisecting the mandible, removing the buccal tissue covering the masseter muscle with a size 15 scalpel blade, and rinsing off blood with 1X PBS. Tissues were either fixed in 4% PFA in 1X PBS overnight and stored in 1X PBS prior to processing and paraffin-embedding, immediately used for RNA extraction or stored in RNALater (ThermoFisher;

#AM7021) at 4°C for use within 2 weeks, or digested in 2U/mL dispase II in KBM-Gold overnight at 4°C while gently rotating for dermal-epidermal separation.

Histology, Immunohistochemical (IHC), and Immunofluorescence (IF) staining

Formalin-fixed, paraffin embedded sections (FFPE) were sectioned at 5μm and hematoxylin & eosin-stained by Histoserv (Germantown, MD). For IHC, 5μm FFPE sections were also stained for Ly-6G-1:100 (ThermoFisher; #16966882) by Histoserv. For IF staining, 5μm FFPE sections were sequentially rehydrated (xylenes, 100%, 95%, 70%, and 50% ethanol) and antigens retrieved with 10mM sodium citrate (pH 6.0) in the Antigen Retriever 2100 (aptum; #R2100-US) for one cycle (~15 minutes) followed by incubation in the heated retriever for 20 minutes. Sections were blocked for 1 hour with 10% BlokHen II (Aves Labs; BH1001), 5% normal goat serum (ThermoFisher; #31872), 0.1% TX-100 (Millipore-Sigma; X100) in 1X PBS. Primary antibodies: PITX1-1:100 (Proteintech; #108721AP) and PCNA-1:100 (Cell Signaling Technology; #13110); both primary antibodies were diluted in blocking buffer. Secondaries used were goat-anti-Rabbit IgG Highly Cross-Adsorbed Alexa Fluor 488 (ThermoFisher; A11034) and Goat-anti-Rabbit IgG Highly Cross-Adsorbed Alexa Fluor 568 (ThermoFisher; A11036); all secondary antibodies were diluted 1:300 in 1X PBS. Slides were incubated with DAPI at 1:1000 in 1X PBS for 5 minutes to stain nuclei. Slides were mounted with ProLong Antifade Gold mountant (ThermoFisher; #P36930) and cured and stored according to manufacturer's specifications prior to imaging. H&E and IHC slides were captured with the Leica Slide Scanner SCN400F and all IF slides captured with a Leica SP8 confocal microscope.

Quantification of Proliferation in Epidermis/Buccal Epithelium

FFPE sections stained for PCNA were imaged at 20X on the Leica SP8 Confocal Microscope. Three random fields from each mouse were captured, and proliferation measured in the tissue by dividing the total number of PCNA+ nuclei in the interfollicular epidermis of the skin samples or

the epithelium of the buccal mucosa by the total DAPI+ nuclei in the interfollicular epidermis/epithelium. Control skin (N = 7), Pitx1+ skin (N = 7), and buccal mucosa (N = 6).

In vivo Wounding Assay

Control and doxycycline-fed adult mice (P98-112) were given 100mg/kg ibuprofen in drinking water two days prior to wounding for analgesia and dorsa shaved one day prior to wounding. Each animal was anesthetized with 2L/minute isoflurane, dorsum swabbed with 70% ethanol, and either a single 6mm or two 3.5mm biopsies were taken by folding the dorsum at the midline and punching ~5mm below the folded midline and between wounds, leaving either two 6mm or four 3.5mm near circular wounds spaced ~1cm apart. Wounds were covered by application of Tegaderm™ occlusive dressing (3M) and allowed to heal for up to twelve days. Tegaderm™ dressing was changed every other day when the wounds were photographed. Wound area was measured in ImageJ and expressed as a percent of the Day 0 wound area. Control and Pitx1+ mice (N = 11 each). Wounds were harvested from euthanized mice by extracting the entire wound with attached subcutaneous fat and muscle to prevent disruption of the wound bed. Tissues were fixed in formalin and processed for paraffin embedding and section as described. All wounds were precisely bifurcated prior to embedding to capture the entire wound bed and wound-adjacent skin at the widest extent of the wound.

Quantification of Wound Re-epithelialization and Granulation

H&E-stained FFPE wound sections were imaged with the Leica Slide Scanner SCN400F to capture the entire wound anatomy. Wound re-epithelization was measured in ImageJ by the distance the epithelial tongue of the epidermis had traveled into the wound bed. Specifically, it was measured by starting from the edge of the wound (histologically apparent by the absence of the panniculus carnosus muscle) and measuring a straight line to the furthest keratinocyte in the wound bed on both sides of the wound. Both sides were averaged to produce a single re-

epithelization distance for each mouse. Granulation tissue was identified histologically and its area measured in ImageJ on both sides of the wound, averaging the two values to produce a single granulation area for each mouse. Control and Pitx1+ mice (N = 6 each).

Primary cell isolation

Primary keratinocytes were isolated and cultured in a protocol adapted from those previously described (2, 3). Neonatal (P1-3) TRE-*Pitx1*/*Krt5*-rtTA pups were euthanized according to NIH IACUC guidelines and rinsed with povidone-iodine, 70% ethanol, and autoclaved Millipore-purified water. Skins were dissected and incubated in 2U/mL dispase II (Millipore-Sigma; #4942078001) diluted in fully supplemented KBM Gold Keratinocyte Growth Basal Media (Lonza; #00192060) while gently rotating overnight at 4°C. Skins were rinsed in 1X PBS (Gibco; #10010023) and the epidermis and dermis separated. The epidermis was carefully stretched basal side down onto clean cell culture dishes under sterile conditions. Skins were gently floated on pre-warmed 0.25% Trypsin-EDTA (Gibco; #25200056) and gently oscillated for 10-15 minutes at room temperature until basal keratinocytes began to dislodge from skin. A volume of soybean trypsin inhibitor (Millipore-Sigma; #10109886001) equal to the volume of trypsin was added to the dish, mixed, and the skins repeatedly rubbed against the plate to further dislodge keratinocytes. Suspended keratinocytes were pooled into conical tubes on ice, and the digested epidermises were further rubbed against the plate bottom after the addition of KBM-Gold and pooling with the first fraction on ice. This procedure was repeated once more before a final washing of the plate with KBM-Gold. The keratinocytes suspension was triturated 50X with a 10mL serological pipette prior to filtering through 100μm cell strainers (Fisher Scientific; #0877119) and centrifugation at 300 *x* g for 5 minutes at 4°C. Pelleted cells were resuspended in supplemented KBM-Gold and were seeded in rat tail Collagen I-coated cell culture dishes (Corning; #354249) at concentrations appropriate for specific experiments. All cells were cultured at 37°C and allowed to adhere

overnight prior to a wash with 1X PBS and replenishment of KBM-Gold for subsequent experiments.

Scratch migration assay

Primary keratinocytes were harvested and seeded at $1x10^6$ cells per well of a 24-well cell culture plate and four wells were each supplemented with either vehicle (PBS) or 2µg/mL doxycycline (Sigma; #10592139) for control and Pitx1+ conditions, respectively. Cells were cultured for 24 hours at 37°C prior to scratching with a 200µL pipette tip held vertically and streaked down the middle of the well. Cells were washed once with 1X PBS and replenished with vehicle/doxycycline-containing KBM-Gold. Three areas of the scratched area were marked and photographed at 0 (initial image), 24, and 48-hours post-scratching in the same areas at 20X (Zeiss Axio Vert.A1). Area of the exposed area was measured in ImageJ and the percentage of scratch array remaining open was normalized the area of the 0-hour area. Four independent experiments were conducted.

RNA isolation from primary keratinocytes and mouse tissue

Total RNA was isolated from primary keratinocytes or whole mouse skin with the RNeasy Mini kit (Qiagen; #74104). Primary keratinocytes were directly scrapped with lysis Buffer QLT and RNA extracted according to manufacturer's instruction. Depilated whole skin was minced into >1mm sections and homogenized with TRIzol (ThermoFisher; #15596026) in 1.4mm ceramic bead tubes (Fisher; #196273) with a Precellys 24 Homogenizer (Bertin; 6500 rpm, 3 x 15 second pulses with 5 second pause between pulses; 5-minute incubation on ice; repeat once more). Chloroform was added the homogenate and shaken vigorously until combined. Homogenate was spun at 12,000 *x g* for 15 minutes at 4°C prior to removing the top aqueous layer to a new tube and continuing the RNeasy extraction protocol according to manufacturer's instruction. Whole

RT-PCR

cDNA was prepared from isolated RNA with the SuperScript III kit (ThermoFisher; 18080093) with random hexamers according to manufacturer's instruction. RT-PCR was performed for *Pitx1* and *Actb* on the StepOnePlus Real-Time PCR System (Applied Biosystems; 4376600) with PowerUP SYBR Green Master Mix (ThermoFisher; A25741). *Pitx1* primers; forward: $^{5'}$TGGAGGCCACGTTCCAAAG$^{3'}$, reverse: $^{5'}$GTTCTTGAACCAGACCCGCAC$^{3'}$. *Actb* primers; forward: $^{5'}$GGCTGTATTCCCCTCCATCG$^{3'}$, reverse: $^{5'}$CCAGTTGGTAACAATGCCATGT$^{3'}$. Data are provided as fold change ($2^{\Delta C_T}$) of *Pitx1* C_T - *Actb* C_T and normalized to the average fold change of the control samples. Control and Pitx1+ mice (N = 6 each) and Control and Pitx1+ primary keratinocytes (N = 6 independently isolated cultures each).

Bulk RNA-seq

Libraries were made from RNA extracted from whole adult murine skin with the NEBNext Ultra II RNA Library Prep Kit (New England Biolabs; E7770L) according to manufacturer's instruction. RNA was analyzed on TapeStation 2200 (Agilent) prior to library prep, keeping samples only with RNA Integrity Numbers (RIN) > 7.5. Amplified libraries were sequenced in the NIAMS IRP Genomics Technology Section with the NovaSeq 6000 (Illumina) and resulting reads processed and analyzed by the NIAMS IRP Biodata Mining and Discovery Section (Bethesda, MD). Expression (RPKM) and fold-changes were calculated and analyzed with Partek Genomics Suite (https://www.partek.com/). Genes that were significantly up- or down-regulated (q < 0.05, FC > 2) between the Control and PITX1+ skins were analyzed with Ingenuity Pathway Analysis software (IPA; Qiagen) to see significantly activated 'Disease & Functions' pathways in IPA (Z-score > 2).

Single-cell RNA-seq

Two 6mm biopsies from Control or PITX1+ skin, pooled fractions of buccal mucosa (each sample had buccal mucosa from ~5 Control mice pooled together), or three pooled 3.5mm Day 4 wounds

from each mouse (excised with 6mm punch biopsies) were minced into >1mm sections and digested with the Whole Skin Dissociation Kit (Miltenyi; 130101540) according to manufacturer's instruction and with adaptations. Briefly, minced tissues were placed in a gentleMACS C-Tube (Miltenyi; 130093237) with 435µL Buffer L, 12.5µL Enzyme P, 50µL Enzyme D, and 2.5µL Enzyme A and incubated for 2.5 hours at 37°C while gently mixing. The digestion was halted by adding 500µL of *unsupplemented* KBM-Gold and the C-Tube inverted, ensuring tissue remains in the liquid around the stator. Tissues were dissociated with the gentleMACS Dissociator (Miltenyi; 130093235) program 'h_skin_01' once. Cells were briefly centrifuged to collect at the bottom of the tube, gently pipetted to resuspend, and filtered sequentially through 70µm and 30µm Pre-Separation Filters (Miltenyi, #130095823 & #130041407, respectively), rinsing each filter with excess KBM-Gold. Cells were pelleted at 300 *x g* for 10 minutes at 4°C and resuspended in 1mL of KBM-Gold and counted for direct submission (unwound skin samples and buccal mucosa; often >90% viability for samples directly after digestion and dissociation) or in 1mL of 0.2% BSA in 1X PBS for live-dead FACS performed by NIAMS IRP Flow Cytometry Section (wound skin).

Up to 50,000 live cells from the unwounded/wound skins and buccal mucosa were submitted to the NIAMS Genomics Technology Section for library processing and sequencing with the Chromium Single Cell 5' workflow (10X Genomics). Libraries were sequenced on the NovaSeq 6000 (Illumina) to generate 151 bp paired-end reads. Reads were aligned, quality controlled, and quantified with Cell Ranger v7.1 (10X Genomics). Processing data for analysis began with identifying ambient RNA contamination and potential doublets with the R packages SoupX and scDblFinder, respectively (4, 5). Subsequent processing was performed with the Seurat *v5* R package (6). Cells with >5% mitochondrial gene, <200 'nFeature_RNA', or >5000 'nFeature_RNA' were removed from the datasets. Unwounded Control and PITX1+ skin and buccal mucosa samples were integrated with the 'HarmonyIntegration' method in Seurat (7). Likewise, wounded Control and PITX1+ skins were integrated similarly. Integrated data sets were annotated manually

based on enriched markers within each cluster at a broad ‘celltype’ level and more specific ‘subtype’ level following ‘celltype’ identification and re-clustering. Additional R packages used for downstream analysis include Monocle3 and MultiNicheNet (8, 9). Ingenuity Pathway Analysis was conducted on cell subtypes based on the set of differentially-expressed genes identified between PITX1+ and Control data sets (Seurat ‘FindMarkers()’ with ‘only.pos = F’, ‘test.use = MAST’, ‘latent.vars = sex’, ‘min.pct = 0.25’, and ‘logfc.threshold = 0.5’). Detailed code for quality control, data processing, and analysis has been deposited to Github (url: ID#). Control mice (N = 8; 154,888 cells), PITX1+ mice (N = 8; 153,330 cells), buccal mucosa pools (N = 7; 71,254 cells).

Pseudotime analysis of keratinocytes and Oral Pseudotime Gene Expression Score

All keratinocytes from the integrated dataset (Control skin, PITX1+ skin, and buccal mucosa) were subsetted into skin-only and oral datasets and re-clustered based on a new uniform manifold approximation and projection (UMAP). Keratinocyte subsets in both datasets were then manually annotated based upon cluster gene enrichment. Skin-only keratinocytes were further subsetted into interfollicular epidermal (IFE) and hair follicle datasets for further analysis. To score the similarity of PITX1+ keratinocyte differentiation to oral keratinocyte differentiation, Monocle3 pseudotime analysis was performed on the skin IFE subset and all oral keratinocytes (excluding ‘Oral Stress Basal’ and ‘Oral Stress Suprabasal’ subtypes). Select genes that were highly expressed in oral keratinocytes were selected to be plotted as a function of pseudotime with the ‘plot_genes_in_pseudotime()’ function for both Control and PITX1+ keratinocytes in the skin-only dataset and the oral dataset. To determine the expression of oral differentiation-associated genes in the skin upon PITX1 expression, the top 100 genes with the highest pseudotime-dependent expression (q-value < 0.05, 100 genes with the highest Moran’s-I statistic) in the oral keratinocyte dataset were identified. The average expression of this set of highly differentiation-dependent oral genes (‘Oral Pseudotime Gene Expression Score’) was determined in the IFE keratinocytes of the skin-only dataset using Seurat ‘AddModuleScore()’ function, and the distributions of scores

between Control and PITX1+ IFE keratinocytes across the ‘Proliferating,’ ‘IFE Basal,’ and ‘IFE Suprabasal’ subtypes were plotted as violin plots.

Xenium *in situ*

5µm FFPE sections were cut in RNAse-free conditions directly onto Xenium slides (10X Genomics) and processed according to manufacturer’s instruction. Slides with sectioned tissues were stored in a desiccator at room temperature until ready to be processed. A customized probe set of 328 genes was used to analyze all tissues (Supplementary Table 1). Slides were processed on the Xenium instrument with Xenium Analyzer v1.6.1 software and the standard segmentation algorithm was used to define cellular areas. Data were analyzed with both Xenium Explorer v2.0.0 (10X Genomics) and the Giotto package (10). Giotto was used to merge the images and transcripts of the separate Xenium runs of healthy Control and PITX1+ skin and buccal mucosa and identify clusters of cells that were manually annotated for cell type and subtypes (based on enriched gene expression) while Xenium Explorer was used to generate images of the cell layer masks and position of identified transcripts. 50,416 Control, 214,767 PITX1+, and 66,389 buccal cells were counted for the healthy tissue data set. Giotto was also used to determine cell cluster markers for the Control and PITX1+ wound section, but no merging or further processing was performed as only the single slide was analyzed. Cell type numbers were normalized against the area of tissue (mm^2) as measured by QuPATH v0.3.2 (11).

CUT&Tag-seq

The entire freshly harvested depilated and defatted dorsal skins from Control and PITX1+ mice were incubated in 10mL of 2U/mL dispase II for 2 hours at 25°C while gently rotating. Skins were rinsed twice in 1X PBS and excess liquid dabbed on Kimwipes prior to spreading the tissue onto dry tissue culture plates, dermis side down. Using a #10 scalpel blade held perpendicularly to the skin, the epidermis was gently scraped from the dermis and pooled in a gentleMACS C-Tube with

500μL of Buffer L. 12.5μL of Enzyme P and 2.5μL of Enzyme A were then added, and the samples were incubated for 1 hour at 37°C while gently rocking. The remaining steps were identical to those described in the 'Single-cell RNA-seq' section above to generate a suspension of single *epidermal* cells. Cells were resuspended in 4mL of *unsupplemented* KBM-Gold for counting. The vast majority (>95%) of the epidermal cell suspensions are keratinocytes, though a small number of melanocytes, epidermis-resident immune cells, and stray fibroblasts likely constitute a minute proportion of the suspension. Control mice (N = 3) and PITX1+ mice (N = 3).

CUT&Tag-seq was performed on epidermal sections from Control and PITX1+ skin using the CUTANA CUT&Tag Kit v1 (EpiCypher; #141102) according to manufacturer's instruction with adaptations (12). 600,000 epidermal cells from each mouse skin were transferred to individual 1.5mL microcentrifuge tubes (need at least 100,000 cells per CUT&Tag reaction). Cells were pelleted at 600 *x g* for 3 minutes at 4°C and resuspended in 1mL of *unsupplemented* KBM-Gold. Cells were gently fixed by adding 6.29μL of 16% formaldehyde, methanol-free (0.1% final concentration; ThermoFisher; #28906) and incubated at room temperature for 1 minute. Formaldehyde was neutralized by adding 67.1μL of 2M glycine (125mM final concentration) and inverted to mix quickly. Cells were pelleted at 600 *x g* for 3 minutes at 4°C and resuspended by vortexing vigorously for 10 seconds in 600μL of Nuclear Extraction Buffer (100μL per reaction). Nuclei were extracted by incubating samples for 30 minutes at 4°C while rotating, vortexing vigorously (10 seconds) every 10 minutes. Nuclei were pelleted at 600 *x g* for 3 minutes at 4°C and resuspended by vortexing vigorously for 10 seconds in 630μL of Nuclear Extraction Buffer (105μL per reaction). 100μL of resuspended nuclei were then aliquoted into the provided 8-strip PCR tubes (~100,000 cells-worth of nuclei per reaction). 1μg of primary antibodies for each reaction: anti-rabbit IgG Negative Control Antibody (EpiCypher; #130042t), PITX1 (Proteintech; #108721AP), H3K4me3 (abcam; #ab213224), H3K27ac (abcam; #ab177178), and H3K27me3 (abcam; #ab192985). K-MetStat Panel (EpiCypher; #191002t) was added to IgG reactions.

Libraries were PCR-amplified with 16 cycles. Library size distribution was analyzed on the TapeStation 2200, most libraries were between 200-700bp in size with the highest peak occurring at ~320-350bp. Samples were sequenced on the NovaSeq 6000 in the NIAMS Genomic Technology Section to generate ~3-12 million total reads per sample. Reads were quality controlled with FastQC, aligned via Bowtie2 and normalized using RPKM, peaks called with SEACR, and differential chromatin occupancy using DESeq2 (13, 14). Visualizations include genomic tracks of IgG, H3K4me3, and Pitx1 peaks using the University of Washington Epigenome Browser (https://epigenomegateway.wustl.edu/browser/) and heatmaps over transcriptional units using the deepTools package (15). Ingenuity Pathway Analysis was conducted on the set of genes identified by DESeq2 that were differentially, significantly enriched (fold enrichment ≥ 2, q < 0.05) for PITX1 binding in the PITX1+ mice CUT&Tag-seq samples compared to Control mice. Motif analysis of PITX1-bound sites was performed with HOMER (v4.11.1) including either intergenic peaks or promoter-adjacent peaks (between -2000 and +200 bp from TSS) (16). Intersection of PITX1 peaks with histone markers was performed with Bedtools 2.31.1.

Statistics

Statistics for *in vitro*, *in vivo*, and histologic experiments were conducted in GraphPad Prism v9.4.1 (https://www.graphpad.com/), statistics for scRNA-seq was conducted in R, statistics for bulk RNA-seq conducted in Partek Genomics Suite, CUT&Tag-seq differentially occupied peaks using DESeq2, and IPA Z-scores were calculated within that software package. All bar charts and box plots are expressed as the mean ± SEM with the individual samples plotted. Statistical differences were assessed with either unpaired 2-tailed Student's t-test for only two samples, two-way ANOVA for comparisons with 3 samples, and repeated measure two-way ANOVA for wound healing assay quantification. Tukey's multiple-comparison *post-hoc* test was used generate P-values for comparisons analyzed via ANOVA. P-values > 0.05 were considered significant (*) while P > 0.01 (**) and P > 0.001 (***).

Significance of scRNA-seq cell proportion plots was determined by proportionality testing and *ad hoc* comparisons of the cell types to derive the fold-change in R with the stats package. Any cell population with $P < 0.01$ and $\log_2$ fold-change ($\log_2$FC) of >1.5 (*), >2(**), or >4(***) were considered significant. For comparison of module scores, a pairwise Wilcoxon Rank Sum test with Benjamini & Hochberg multiple comparisons correction was conducted with the R package *stats*. Comparisons were either highly significant ($P \ll 0.001$) or were not significant.

Significantly and differentially-expressed genes from bulk RNA-seq experiments were identified by calculating RPKM values and using 2-way ANOVA in the Partek Genomics Suite. Genes were considered significantly up- or down-regulated if they had RPKM >1, q-value <0.05, and a $\log_2$ fold-change >2. For analysis of genomic peak differential binding in DESeq2, the normalized average peak counts that were identified to be close to annotated genes were compared with DESeq2 differential analysis. Genes with an adjusted P-value >0.05 and a $\log_2$ fold-change >1.5 were considered significant. The sets of genes passing the parameters set for either RNA-seq or CUT&Tag-seq would be fed into IPA for subsequent analysis of pathway activation as measured by Z-scores. For both ‘Disease and Functions’ and ‘Canonical Pathways’ networks in IPA, a pathway was considered activated or suppressed if the Z-score was >2 or <-2, respectively.

REFERENCES

1. Pandey SN, Cabotage J, Shi R, Dixit M, Sutherland M, Liu J, et al. Conditional over-expression of PITX1 causes skeletal muscle dystrophy in mice. *Biol Open.* 2012;1(7):629-39.
2. Lichti U, Anders J, and Yuspa SH. Isolation and short-term culture of primary keratinocytes, hair follicle populations and dermal cells from newborn mice and keratinocytes from adult mice for in vitro analysis and for grafting to immunodeficient mice. *Nat Protoc.* 2008;3(5):799-810.
3. Li F, Adase CA, and Zhang LJ. Isolation and Culture of Primary Mouse Keratinocytes from Neonatal and Adult Mouse Skin. *J Vis Exp.* 2017(125).
4. Young MD, and Behjati S. SoupX removes ambient RNA contamination from droplet-based single-cell RNA sequencing data. *Gigascience.* 2020;9(12).
5. Germain PL, Lun A, Garcia Meixide C, Macnair W, and Robinson MD. Doublet identification in single-cell sequencing data using scDblFinder. *F1000Res.* 2021;10:979.
6. Hao Y, Stuart T, Kowalski MH, Choudhary S, Hoffman P, Hartman A, et al. Dictionary learning for integrative, multimodal and scalable single-cell analysis. *Nat Biotechnol.* 2024;42(2):293-304.
7. Korsunsky I, Millard N, Fan J, Slowikowski K, Zhang F, Wei K, et al. Fast, sensitive and accurate integration of single-cell data with Harmony. *Nat Methods.* 2019;16(12):1289-96.
8. Browaeys R, Gilis J, Sang-Aram C, Bleser PD, Hoste L, Tavernier S, et al. MultiNicheNet: a flexible framework for differential cell-cell communication analysis from multi-sample multi-condition single-cell transcriptomics data. *bioRxiv.* 2023:2023.06.13.544751.
9. Trapnell C, Cacchiarelli D, Grimsby J, Pokharel P, Li S, Morse M, et al. The dynamics and regulators of cell fate decisions are revealed by pseudotemporal ordering of single cells. *Nat Biotechnol.* 2014;32(4):381-6.

10. Dries R, Zhu Q, Dong R, Eng CL, Li H, Liu K, et al. Giotto: a toolbox for integrative analysis and visualization of spatial expression data. *Genome Biol.* 2021;22(1):78.

11. Bankhead P, Loughrey MB, Fernandez JA, Dombrowski Y, McArt DG, Dunne PD, et al. QuPath: Open source software for digital pathology image analysis. *Sci Rep.* 2017;7(1):16878.

12. Kaya-Okur HS, Wu SJ, Codomo CA, Pledger ES, Bryson TD, Henikoff JG, et al. CUT&Tag for efficient epigenomic profiling of small samples and single cells. *Nat Commun.* 2019;10(1):1930.

13. Love MI, Huber W, and Anders S. Moderated estimation of fold change and dispersion for RNA-seq data with DESeq2. *Genome Biol.* 2014;15(12):550.

14. Langmead B, and Salzberg SL. Fast gapped-read alignment with Bowtie 2. *Nat Methods.* 2012;9(4):357-9.

15. Ramirez F, Ryan DP, Gruning B, Bhardwaj V, Kilpert F, Richter AS, et al. deepTools2: a next generation web server for deep-sequencing data analysis. *Nucleic Acids Res.* 2016;44(W1):W160-5.

16. Heinz S, Benner C, Spann N, Bertolino E, Lin YC, Laslo P, et al. Simple combinations of lineage-determining transcription factors prime cis-regulatory elements required for macrophage and B cell identities. *Mol Cell.* 2010;38(4):576-89.

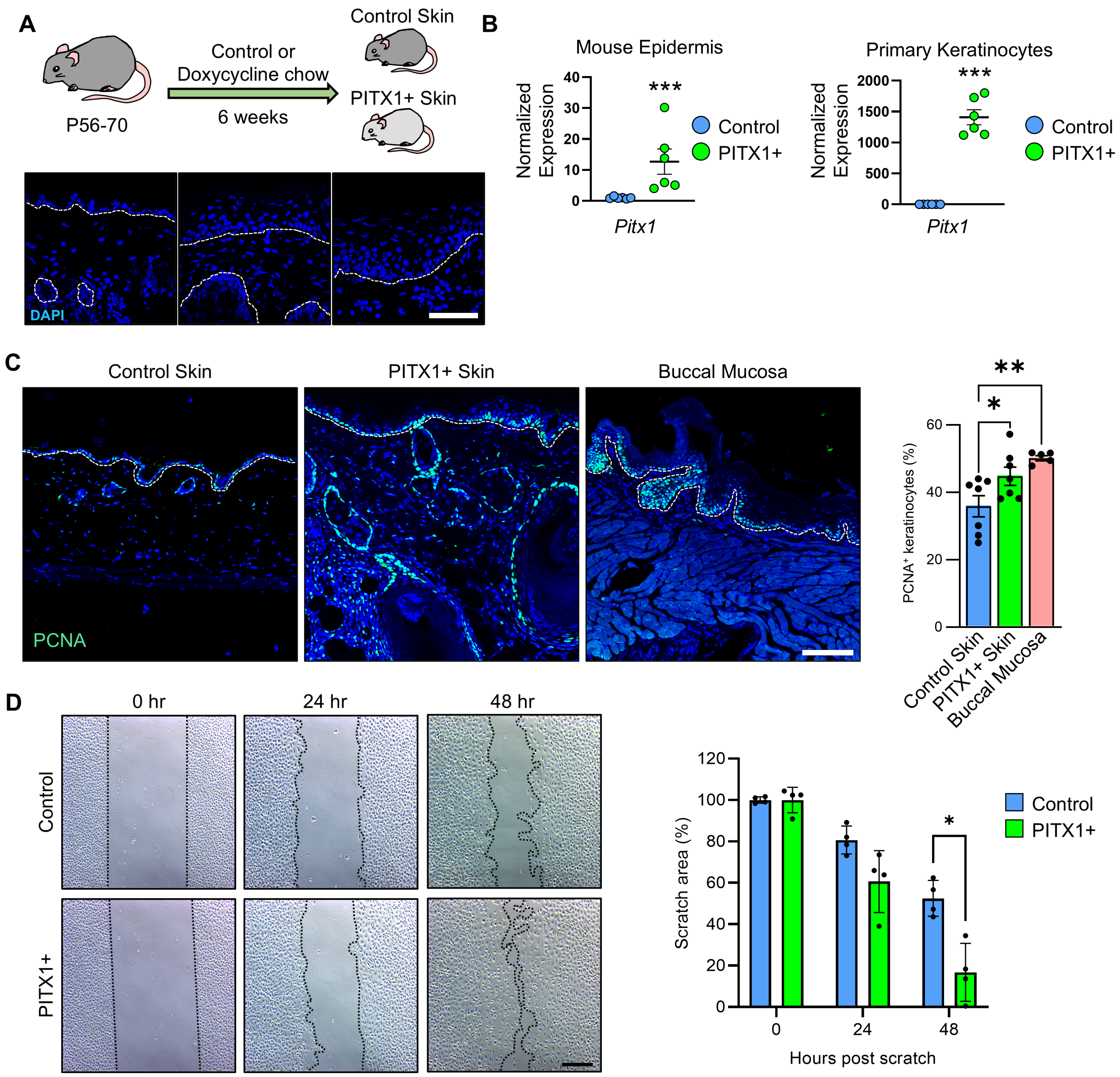

Supplemental Figure 1: *Ectopic PITX1 expression in cutaneous keratinocytes promotes their proliferation and migration.* (A) Schematic of PITX1 induction in TRE-*Pitx1*/*Krt5*-rtTA mice (top) and DAPI for PITX1 IF's in Figure 1B (bottom). (B) qPCR for *Pitx1* in Control (N = 6) and PITX1+ (N = 6) dispase-separated mouse epidermal RNA (left) and vehicle-treated (N = 6) and doxycycline-treated (N = 6) primary keratinocytes from TRE-*Pitx1*/*Krt5*-rtTA neonates (right), both plotted as average ± SEM. Significance was determined by an unpaired Student's t-test (***P < 0.001). (C) Representative IF for PCNA (green) and nuclei (blue) in male Control skin (N = 7), PITX1+ skin (N = 7), and control buccal mucosa (N = 5) to measure IFE keratinocyte proliferation, plotted as average ± SEM (left). Line denotes dermal-epidermal junction. Scale bar = 100 μm. Quantification of PCNA+ nuclei in the IFE in each sample (right). Significance was determined by a one-way ANOVA with Tukey's *post-hoc* testing (*P < 0.05, **P < 0.01). (D) Scratch assay of TRE-*Pitx1*/*Krt5*-rtTA neonatal primary keratinocytes treated with vehicle or doxycycline plotted as average ± SEM (left). Quantification of scratched area remaining open at 24- and 48-hours post-scratching (right). Scale bar = 50 μm. Significance was determined by a repeated-measures two-way ANOVA with Tukey's *post-hoc* testing (*P < 0.05).

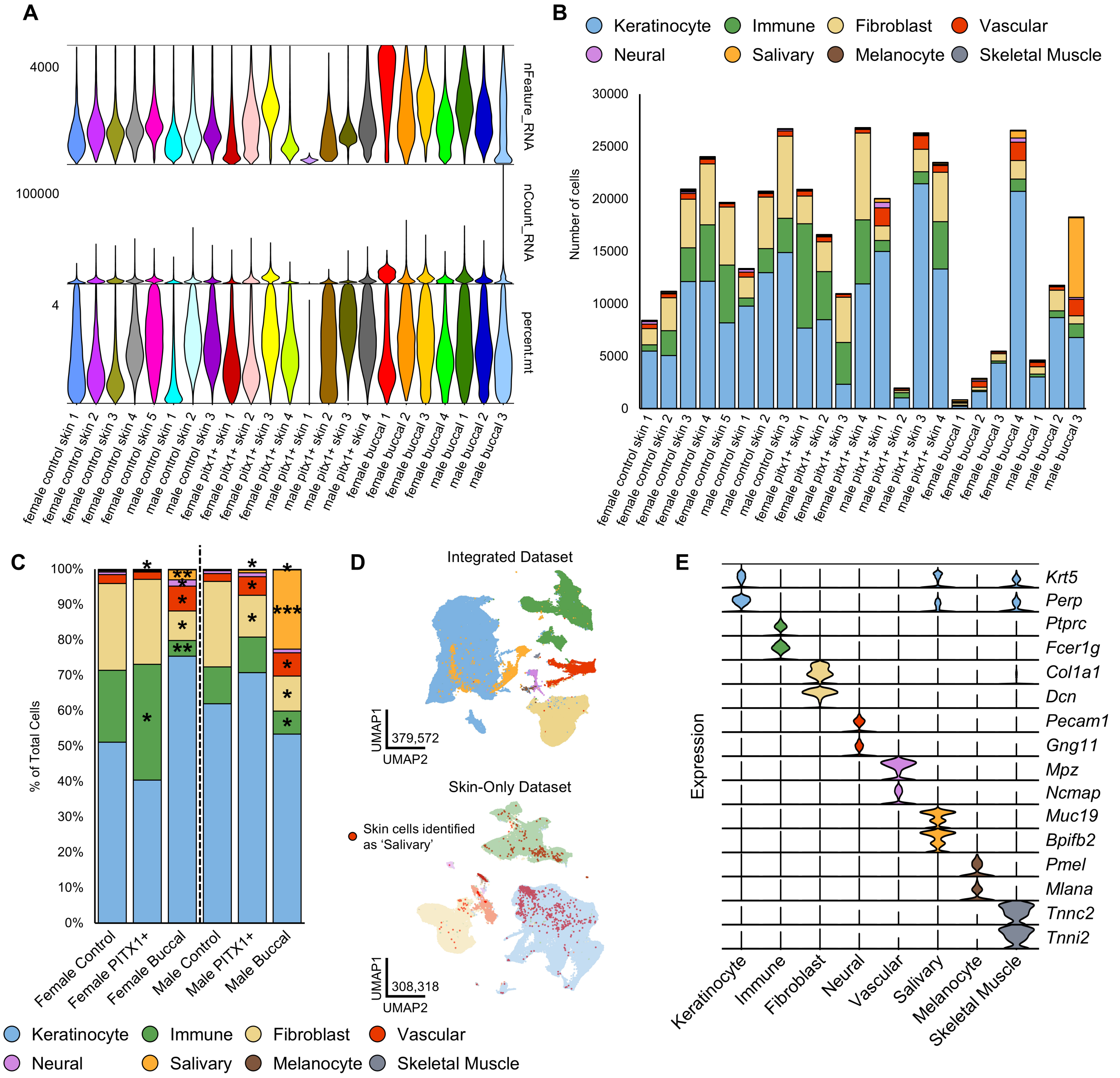

Supplemental Figure 2: *Quality control metrics for healthy tissue scRNA-seq experiments.* (A) Violin plots showing distribution of *nFeature_RNA*, *nCount_RNA*, and *percent.mt* mitochondrial transcripts in cells of male and female Control skin (N = 8), PITX1+ skin (N = 8), and buccal mucosa (N = 7 pools) scRNA-seq samples. (B) Bar charts showing cumulative cell types collected from each scRNA-seq sample. (C) Proportion plot of cell types in each condition and sex. (D) UMAP of integrated Control, PITX1+, and buccal scRNA-seq samples with cell types colored (379,572 total cells, top). UMAP of subsetted skin-only dataset with cells identified as 'Salivary' in integrated dataset highlighted in red (bottom). (E) Violin plots showing representative gene marker expression used to manually annotate each cell type within the scRNA-seq data set. Significance for proportion plots was assessed by proportionality testing followed by *ad hoc* comparisons against the corresponding cell type and the sex-specific Control Skin samples to derive $\log_2$ fold-change ($\log_2$FC) (*P < 0.01 & $\log_2$FC > |1.5|, ***P < 0.01 & $\log_2$FC > |4|).

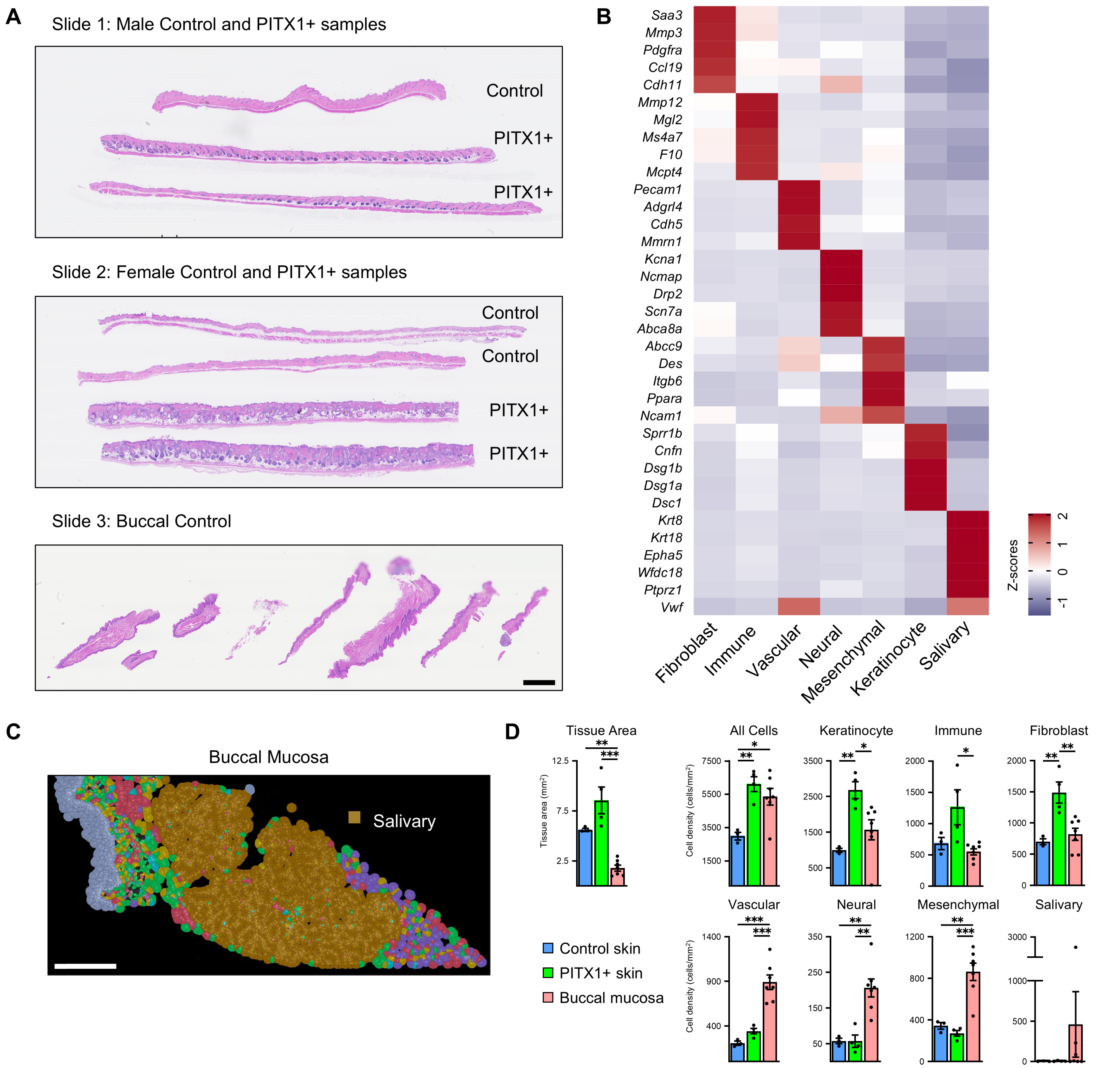

Supplemental Figure 3: *Quality control metrics for healthy tissue Xenium experiments.* (A) H&E images of FFPE sections used for Xenium analysis. Scale bar = 1 mm. (B) Heatmap showing localization of gene marker expression to manually annotate each cell type within the Xenium data set. Z-score is metric of the number of transcripts of a given gene enriched within the boundaries of a cell type; positive Z-score = transcripts of a gene are highly enriched in cells of a given cell type. (C) Example buccal mucosa Xenium section with a large salivary gland (bronze). Scale bar = 200 µm. (D) Tissue area (mm^2) of Xenium sections for Control skin (N = 3), PITX1+ skin (N = 4), and buccal mucosa (N = 7) plotted as average ± SEM (left). Density of cells and all cell types (average cells/mm^2 ± SEM) (right). Significance was determined by a two-way ANOVA with Tukey's *post-hoc* testing (*P < 0.05, **P < 0.01, ***P < 0.001).

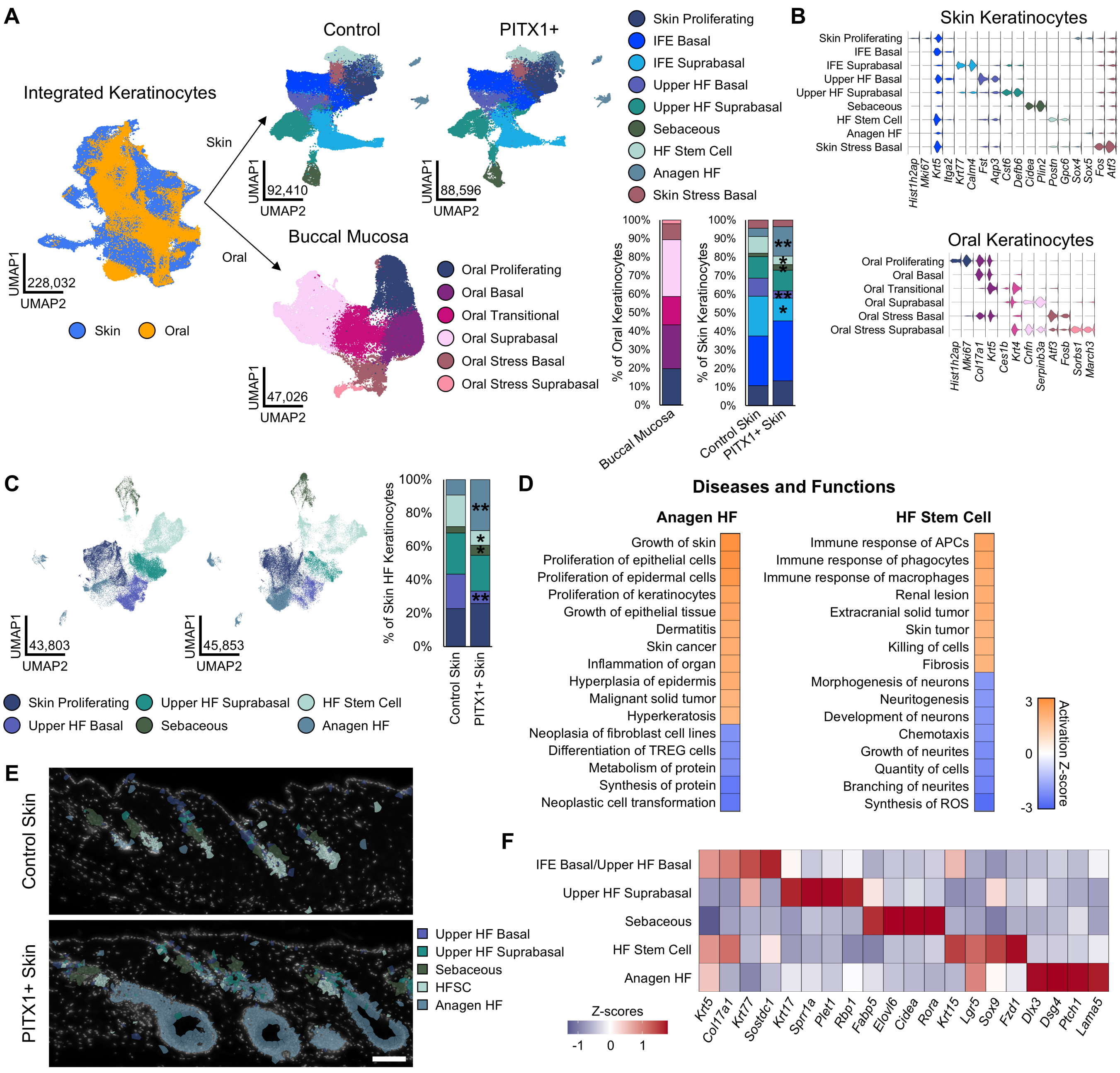

Supplemental Figure 4: *Keratinocyte scRNA-seq data.* (A) UMAP of integrated Control (N = 8), PITX1+ (N = 8), and buccal (N = 7) keratinocytes (left) subsetted into skin-only and oral-only data sets (right). Cells colored by keratinocyte subtypes identified separately in skin and oral tissues. Proportion plots for keratinocyte subtype in each condition. (B) Violin plots showing representative gene marker expression used to manually annotate each keratinocyte subtype. (C) UMAPs of skin-only Control and PITX1+ hair follicle (HF) keratinocyte subpopulations (left). Proportion plot of HF keratinocyte subpopulations (right). (D) Ingenuity Pathway Analysis (IPA) 'Diseases and Functions' ontology of differentially-expressed genes between PITX1+ and Control HF subtypes indicated. Z-score > |2| were considered significantly activated or repressed. (E) Representative Xenium of male Control and PITX1+ skin with HF subtypes labeled. Scale bar = 100 μm. (F) Gene marker expression within the Xenium data to annotate each HF subtype shown in Supplemental Figure 4E. Significance for all proportion plots was assessed by proportionality testing followed by *ad hoc* comparisons against the corresponding cell type in Control skin to derived $\log_2$ fold-change ($\log_2$FC) (*$P < 0.01$ & $\log_2FC > |1.5|$, **$P < 0.01$ & $\log_2FC > |2|$).

A

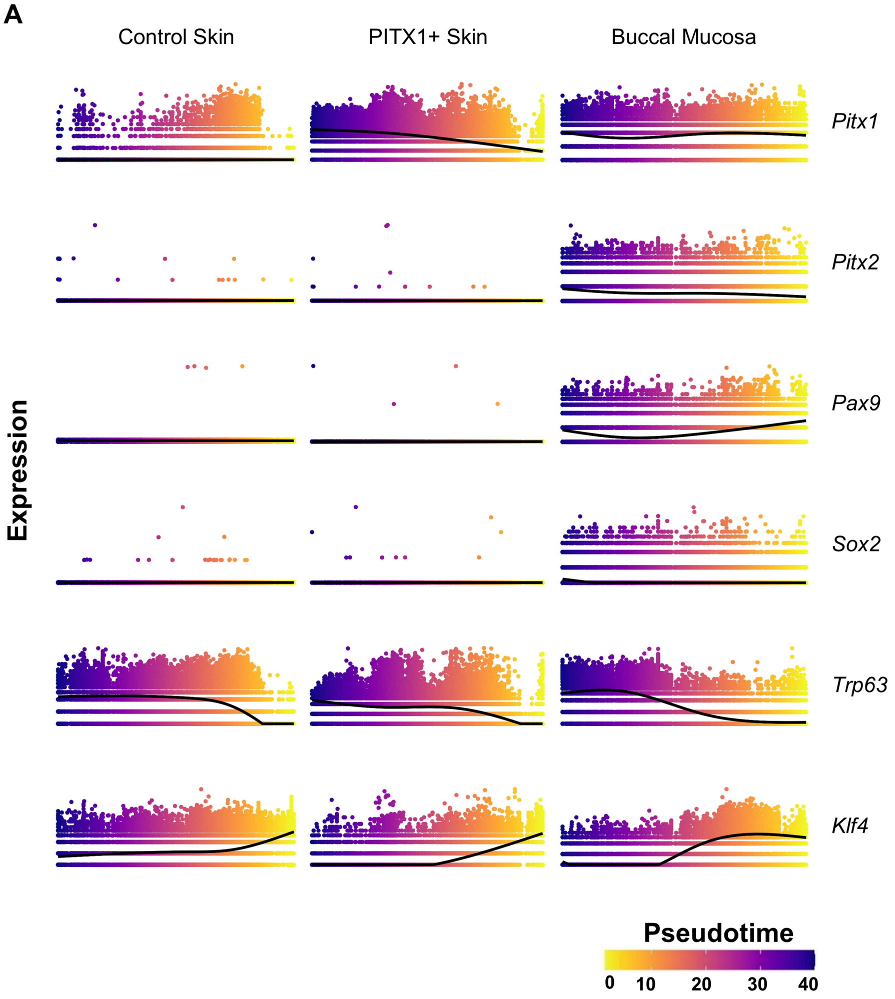

Supplemental Figure 5: *Pseudotime analysis of IFE keratinocytes.* (A) Plots illustrating gene expression as a function of pseudotime of individual cells (dots) in each condition. Line indicates average gene expression over pseudotime.

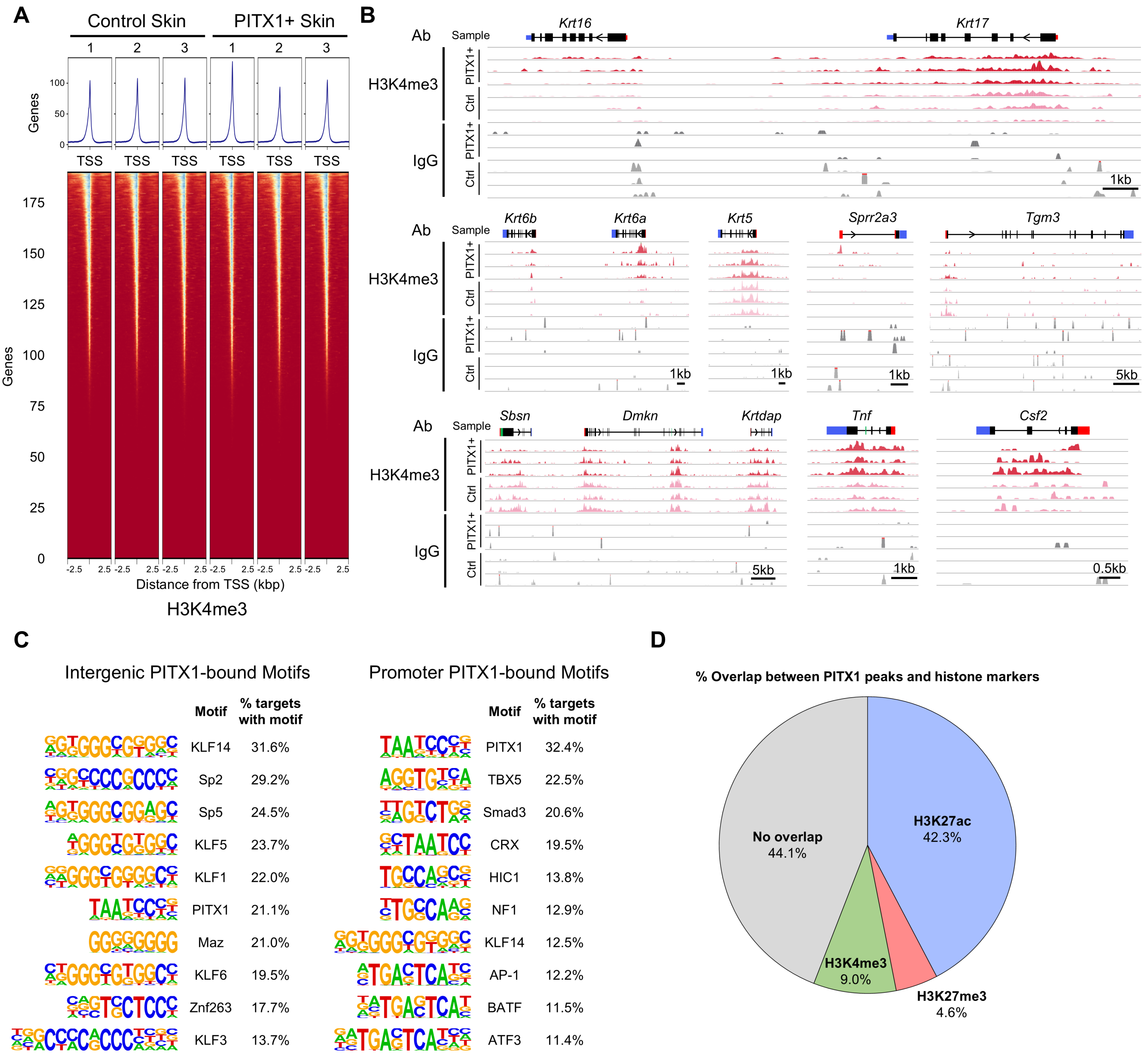

Supplemental Figure 6: *CUT&Tag-seq of H3K4me3 in Control and PITX1+ epidermal cells*. (A) Control (N = 3) and PITX1+ (N = 3) epidermal cell CUT&Tag-seq heatmaps of average genomic occupancy of histone H3K4me3 over transcriptional units, starting 2.5kb upstream of transcription start sites (TSS) and ending 2.5kb downstream of transcription end sites (TES). 'Genes' scale bar on right indicates how many genes have a particular occupancy pattern observed in the heatmap. (B) Representative genomic tracks of Control and PITX1+ epidermal CUT&Tag-seq for H3K4me3 and IgG antibodies. Gene 5'UTR (red bar), exons (black bars), alternatively spliced exons (green bars), 3'UTR (blue bars), and direction of transcription (arrows) represented above tracks. (C) Representative transcription factor motifs enriched for PITX1 binding in intergenic and promoter-proximal (-2kb to +200bp around TSS) regions as identified by Homer. All motifs were significantly enriched (Benjamini q-value << 0.01). (D) Percent of PITX1-bound peaks that overlap areas enriched with histone markers for enhancers (H3K27ac), heterochromatin (H3K27me3), promoters (H3K4me3), and no overlap with assessed histone markers.

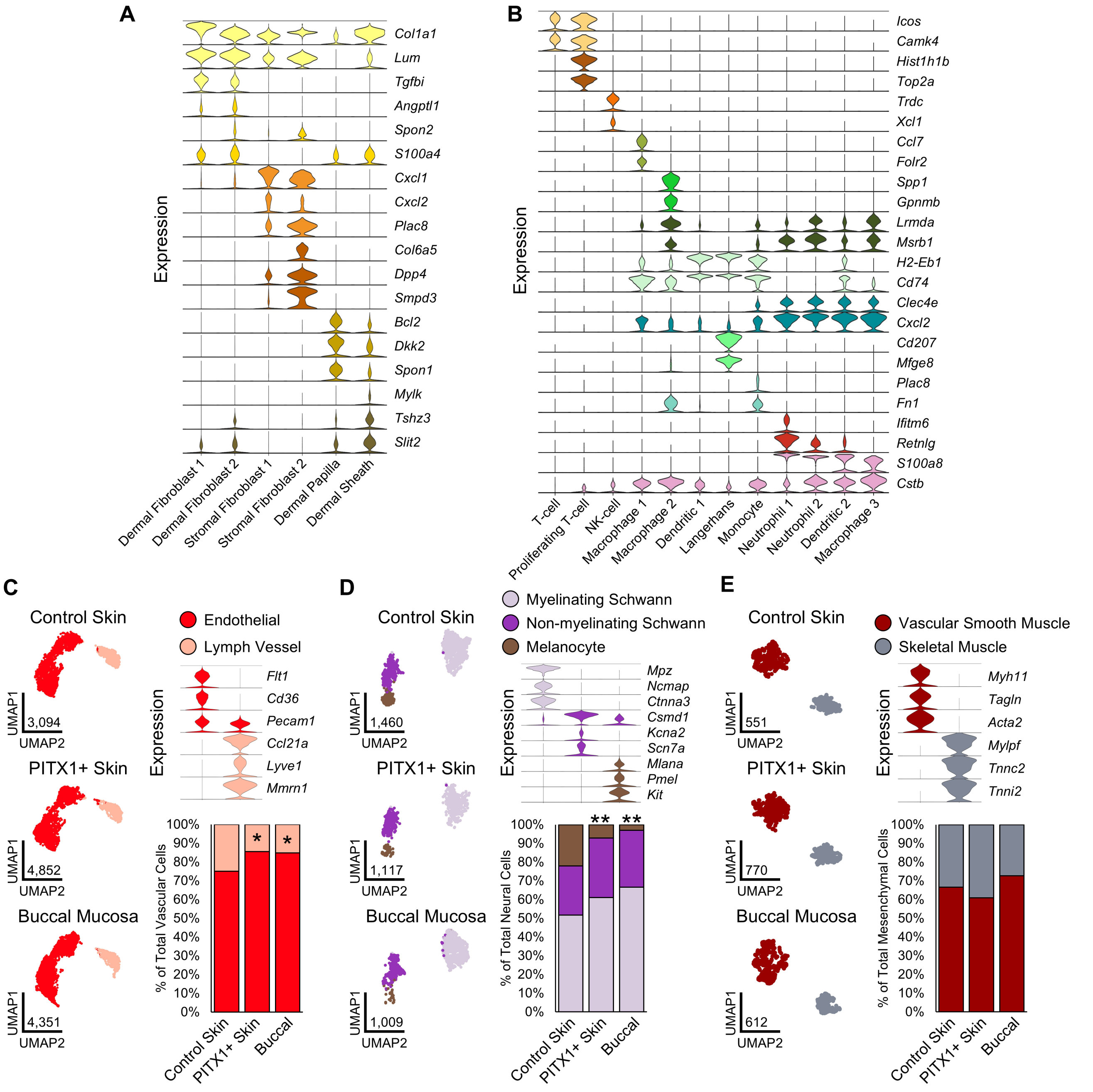

Supplemental Figure 7: *Fibroblast, immune, vascular, neural, and muscle cell scRNA-seq data.* (A) Violin plots showing representative gene marker expression used to manually annotate each fibroblast subtype within the fibroblast scRNA-seq subset. (B) Violin plots showing representative gene marker expression used to manually annotate each immune subtype within the immune cell scRNA-seq subset. (C) UMAPs of Control (N = 8), PITX1+ (N = 8), and buccal (N = 7) vascular cell subpopulations (left). Violin plots showing representative gene marker expression used to manually annotate each vascular subtype and proportion plot of vascular subpopulations (right). (D) UMAPs of Control, PITX1+, and buccal neural cell subpopulations (left). Violin plots showing representative gene marker expression used to manually annotate each neural subtype and proportion plot of neural cell subpopulations (right). (E) UMAPs of Control, PITX1+, and buccal muscle cell subpopulations (left). Violin plots showing representative gene marker expression used to manually annotate each muscle subtype and proportion plot of muscle cell subpopulations (right). Significance for proportion plots was assessed by proportionality testing followed by *ad hoc* comparisons against the corresponding cell type in Control skin to derived $\log_2$ fold-change ($\log_2$FC) (*$P < 0.01$ & $\log_2FC > |1.5|$, **$P < 0.01$ & $\log_2FC > |2|$).

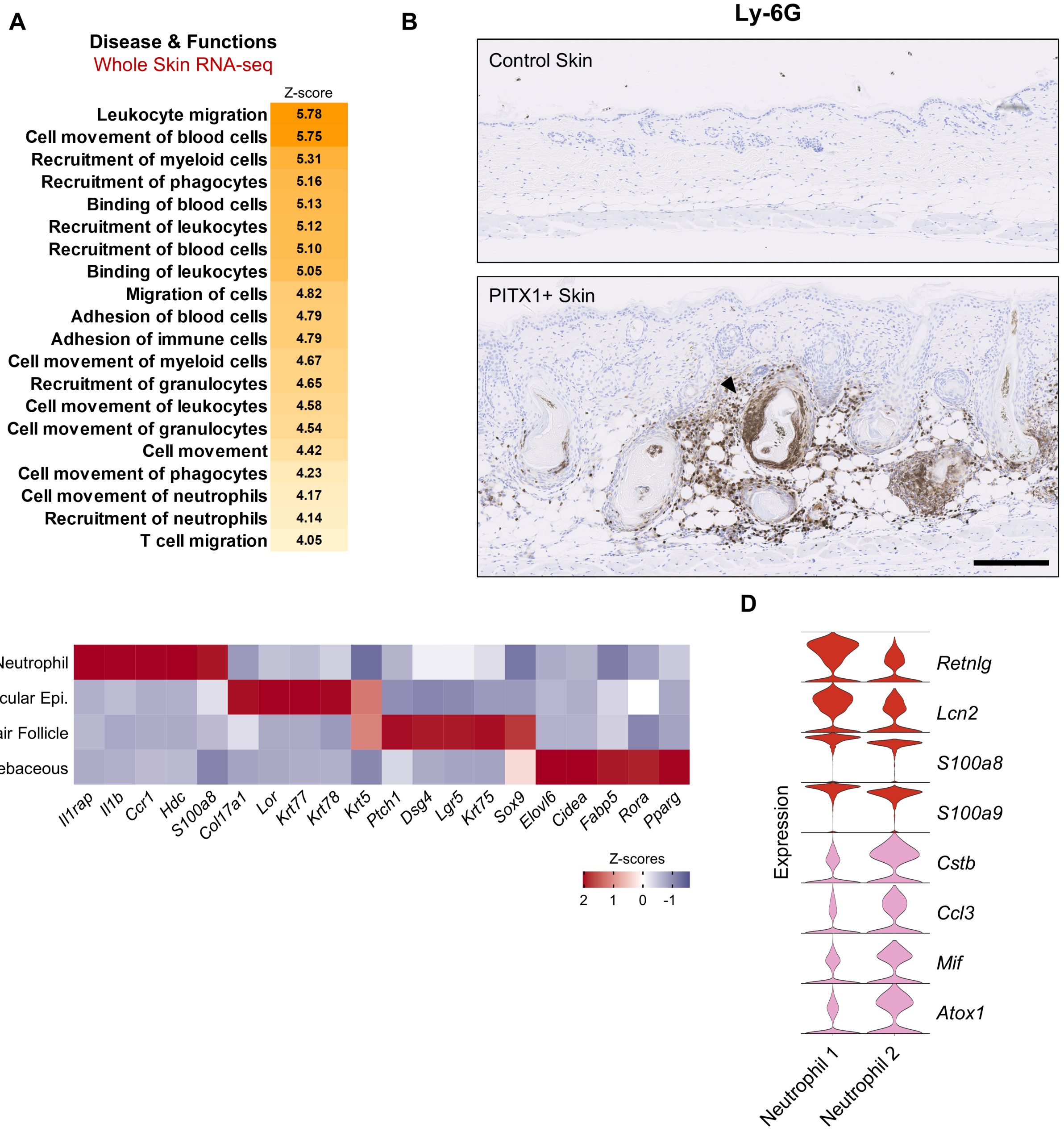

Supplemental Figure 8: *Neutrophils are recruited PITX1+ skin*. Ingenuity Pathway Analysis (IPA) 'Disease and Functions' ontology derived from the differentially-expressed genes between Control (N = 4) and PITX1+ (N = 4) whole skin. Z-score > |2| were considered significantly activated or repressed. (B) Representative IHC for the neutrophil marker Ly-6G in male Control and PITX1+ skin. Arrow points to hair follicle infiltrated by neutrophils. Scale bar = 200 µm. (C) Heatmap showing localization of gene marker expression to manually annotate each cell subtype within the Xenium data shown in Figure 4C. (D) Violin plots showing representative gene marker expression used to manually annotate each neutrophil subtype in Figure 4D.

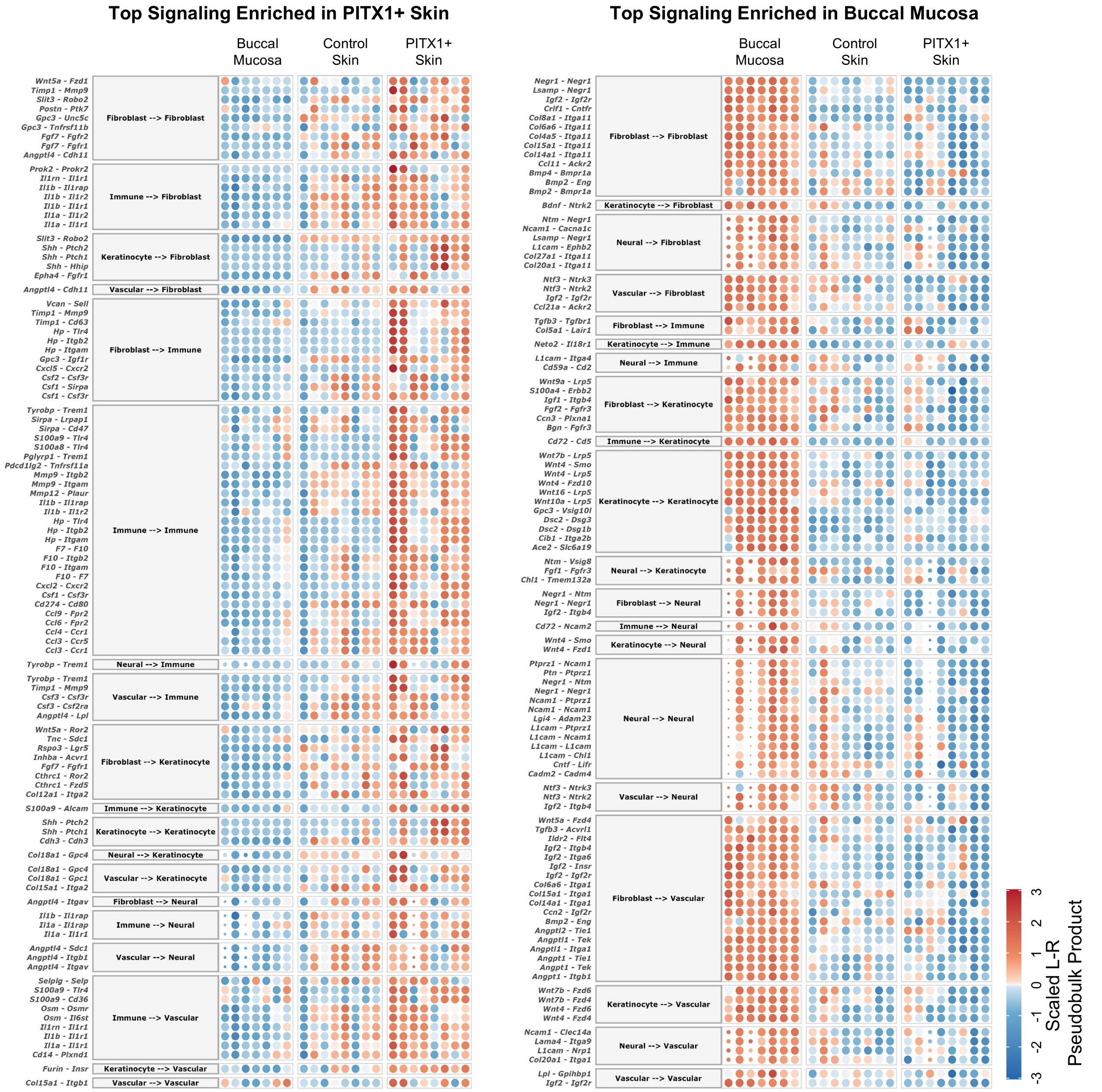

Supplemental Figure 9: *MultiNicheNet predicted ligand-receptor pairs.* Dot plots showing scaled ligand-receptor (L-R) pseudobulk cross product expression of top 100 predicted pairs of ligands (expressed in 'sender' cell types) engaging receptors (expressed in 'receiver' cell types). Left, chart of L-R pairs predicted to be enriched in PITX1+ skin compared to Control skin (related to Figure 5A-B). Right, chart of L-R pairs predicted to be enriched in buccal mucosa compared to Control skin (related to Figure 5C-D).

A

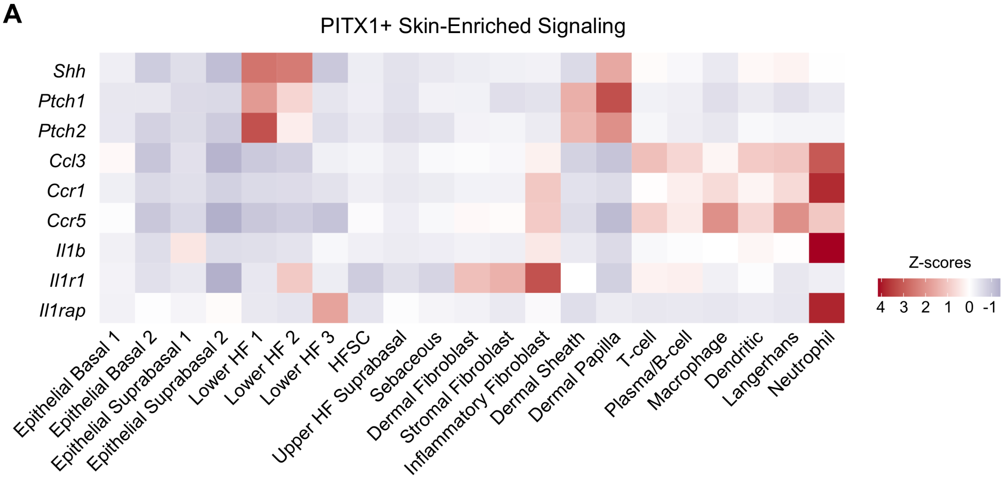

B

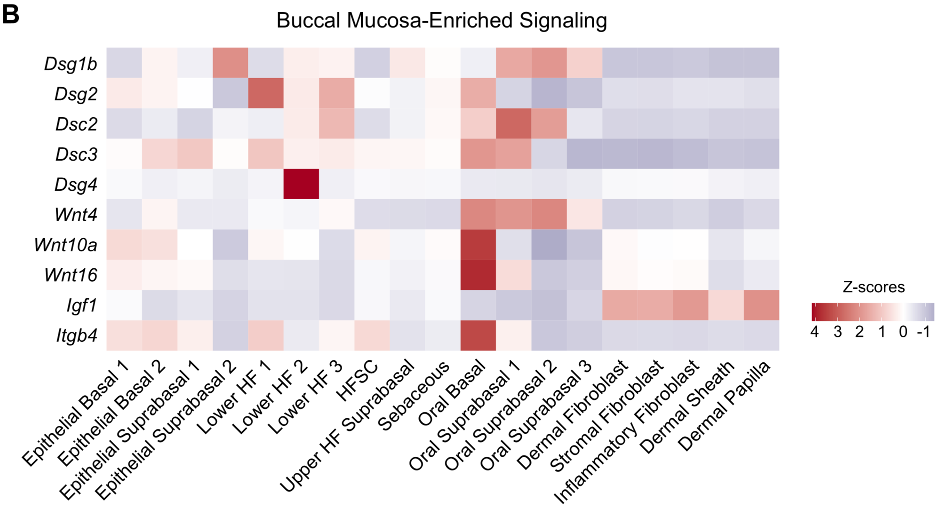

Supplemental Figure 10: *Xenium expression of predicted ligands and receptors identified by MultiNicheNet.* (A) Heatmap showing localization of ligand and receptor genes in Xenium data that were predicted to be more highly active in PITX1+ skin over Control skin by MultiNicheNet analysis in Figure 5A-B. (B) Heatmap showing localization of ligand and receptor genes in Xenium data that were predicted to be more highly active in PITX1+ skin over Control skin by MultiNicheNet analysis in Figure 5C-D.

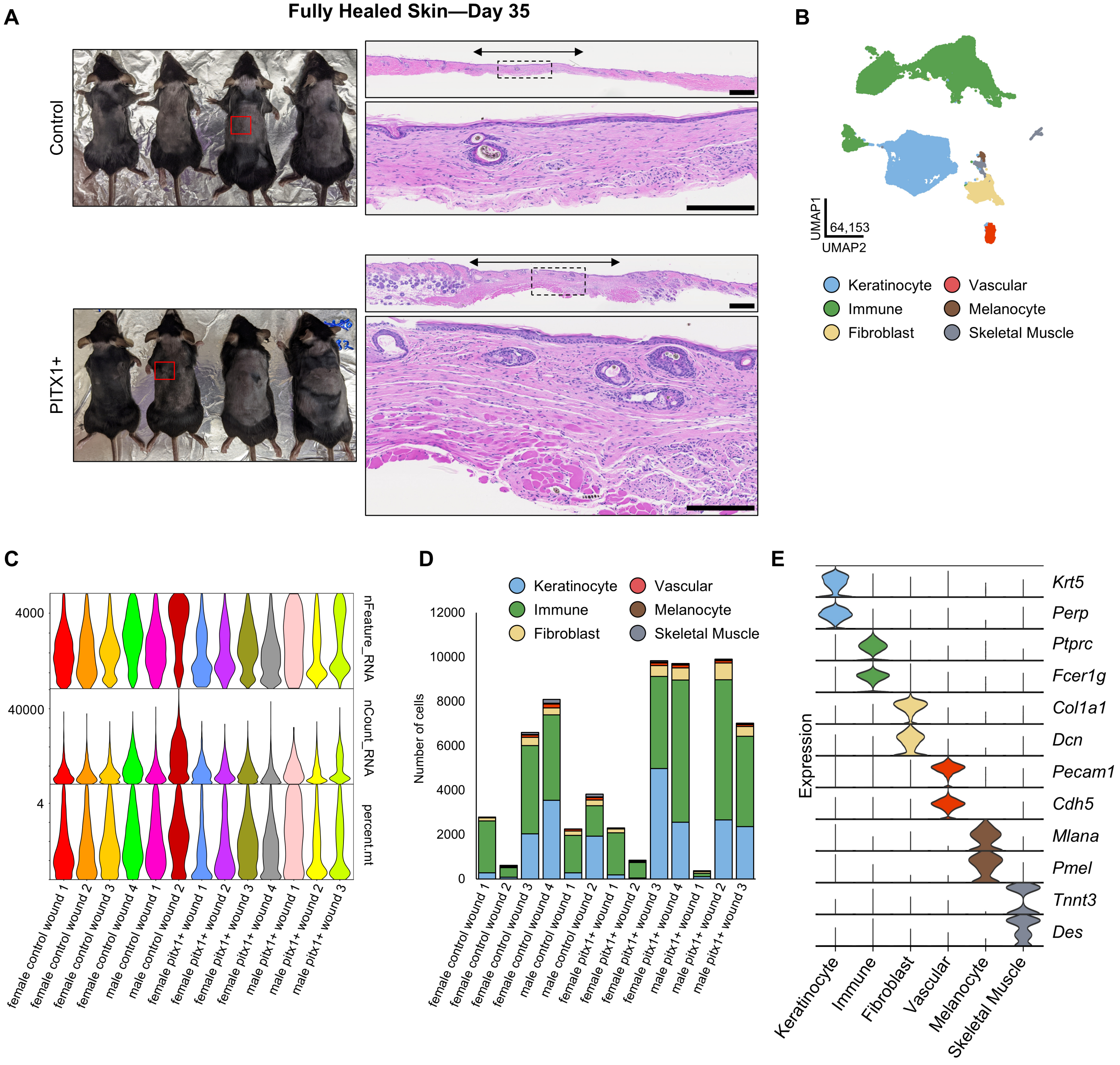

Supplemental Figure 11: *Healed mice and quality control metrics for wound skin scRNA-seq experiments.* (A) Shaved mice dorsa of Control and PITX1+ Day 35 fully-healed wounds (left). Red box indicates area of a single wound analyzed histologically. 2.5x (top) and 20x (bottom) H&E of indicated wound areas (right). Dashed box in 2.5x H&E indicates area enlarged in 20x images. Double-arrowed lines in 20x images show extent of healed wound beds. Scale bars: 2.5x = 500 μm; 20x = 200μm.(B) UMAP of integrated Control and PITX1 wounds with cell types colored (64,153 total cells). (C) Violin plots showing distribution of *nFeature_RNA*, *nCount_RNA*, and *percent.mt* mitochondrial transcripts in cells of male and female Control (N = 6) and PITX1+ (N = 7) wound scRNA-seq samples. (D) Bar charts showing cumulative cell types collected from each scRNA-seq sample. (E) Violin plots showing representative gene marker expression used to manually annotate each cell type within the wound scRNA-seq data set.

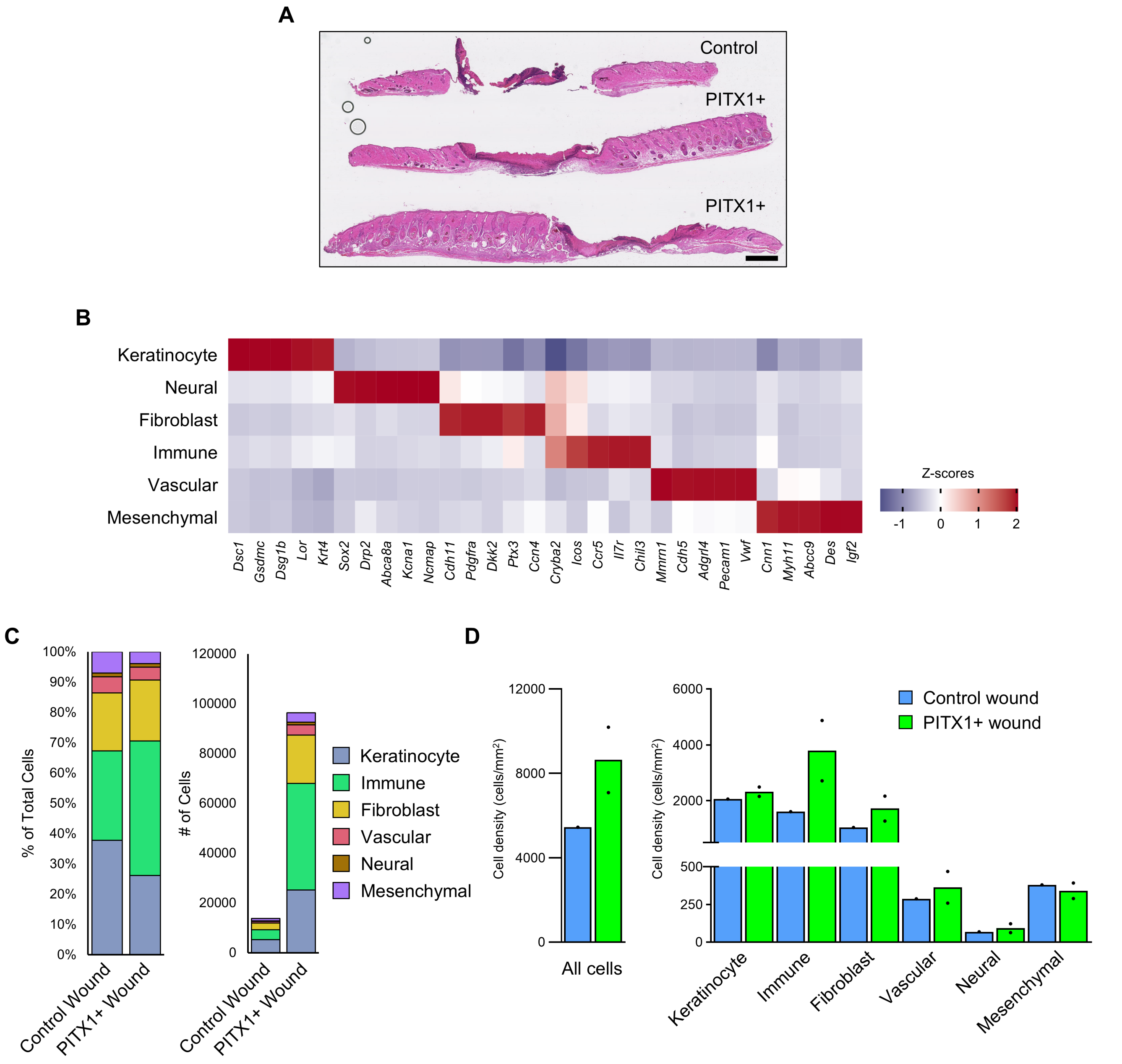

Supplemental Figure 12: *Quality control metrics for wound skin Xenium experiments.* (A) H&E images of FFPE section used for Xenium analysis; male Control wounds (N = 1) and PITX1+ wounds (N = 2). Scale bar = 1 mm. (B) Heatmap showing localization of gene marker expression to manually annotate each cell type within the wound skin Xenium data set. (C) Proportion plot (left) and total number (right) of cell types in each wound skin condition. (D) Tissue area (mm^2) of Xenium sections for Control and PITX1+ wounds plotted as average (left). Density of all cells and cell types (average cells/mm^2) (right). Significance could not be assessed as Control wound had one sample.

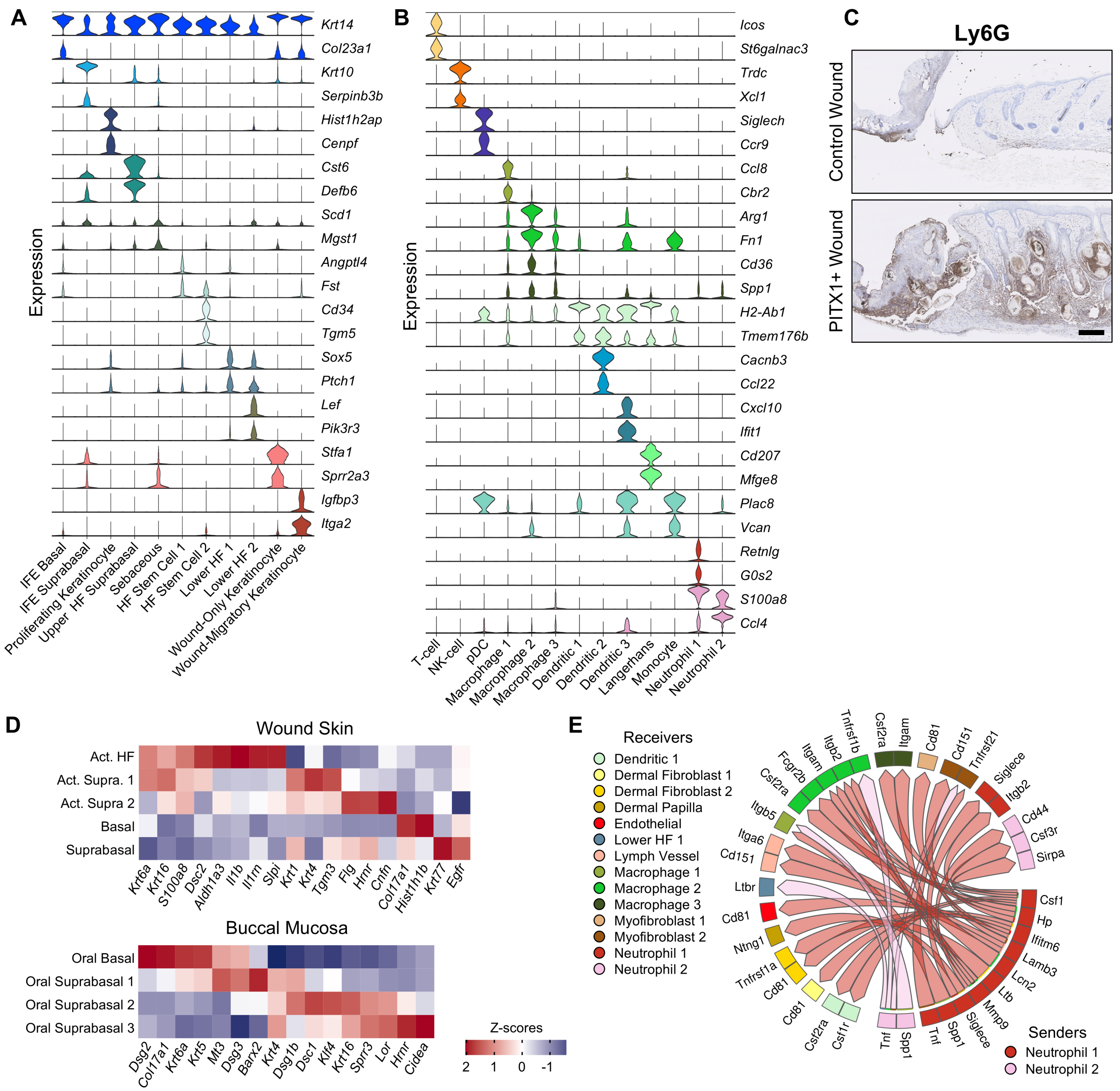

Supplemental Figure 13: *Wound skin Xenium cell subtypes and neutrophil recruitment to wounds.* (A) Violin plots showing representative gene marker expression used to manually annotate each keratinocyte subtype within the wound scRNA-seq data. (B) Violin plots showing representative gene marker expression used to manually annotate each immune subtype within the wound scRNA-seq data. (C) Representative IHC for the neutrophil marker Ly-6G in male Control and PITX1+ wound-adjacent skin. Scale bar = 200 μm. (D) Heatmap showing localization of gene marker expression to manually annotate each cell subtype within the wound skin Xenium data set shown in Figure 6B & D. (E) Circos plot showing the top 20 PITX1+ wound-enriched ligand-receptor (L-R) interactions predicted by MultiNicheNet of originating from neutrophil subtypes. Control (N = 7) and PITX1+ (N = 6).
